# Supplementary material for: Systematic segmentation method based on PCA of image hue features for white blood cell counting
Source: PLoS One. 2021 Dec 31;16(12):e0261857. doi: 10.1371/journal.pone.0261857 (PMC8719728; doi:10.1371/journal.pone.0261857)
Supplement: S1 Appendix — (DOCX) [file pone.0261857.s006.docx]

**S1 Appendix**


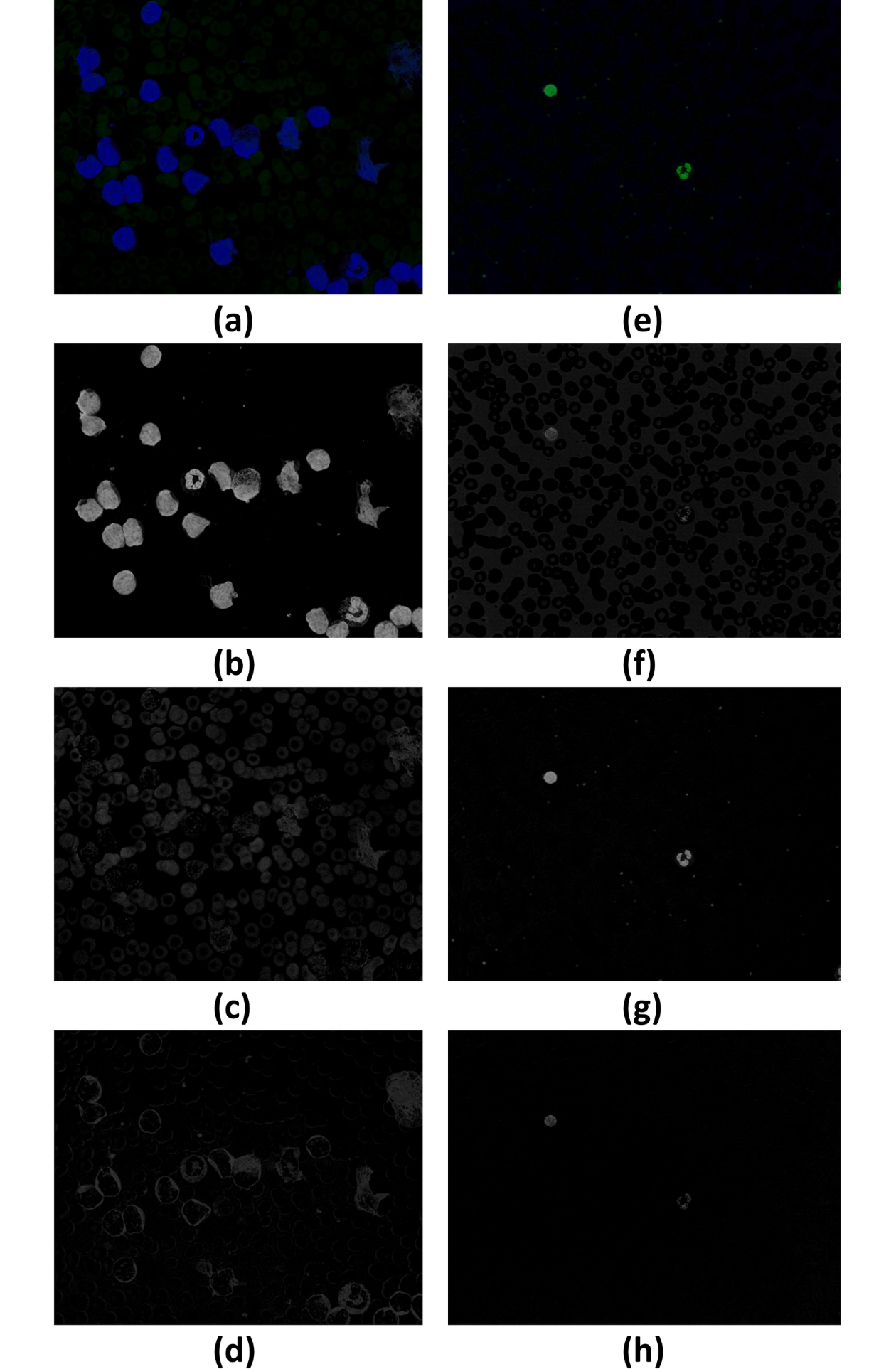


Fig 4. Images obtained by processing the chromaticity in the RGB space of images of Fig 1. Images (a) and (e) were obtained after projecting the colors onto the eigenspace, and images (b) and (f), (c) and (g), and (d) and (h) resulted from projecting the colors with the first, second, and third PCs, respectively.


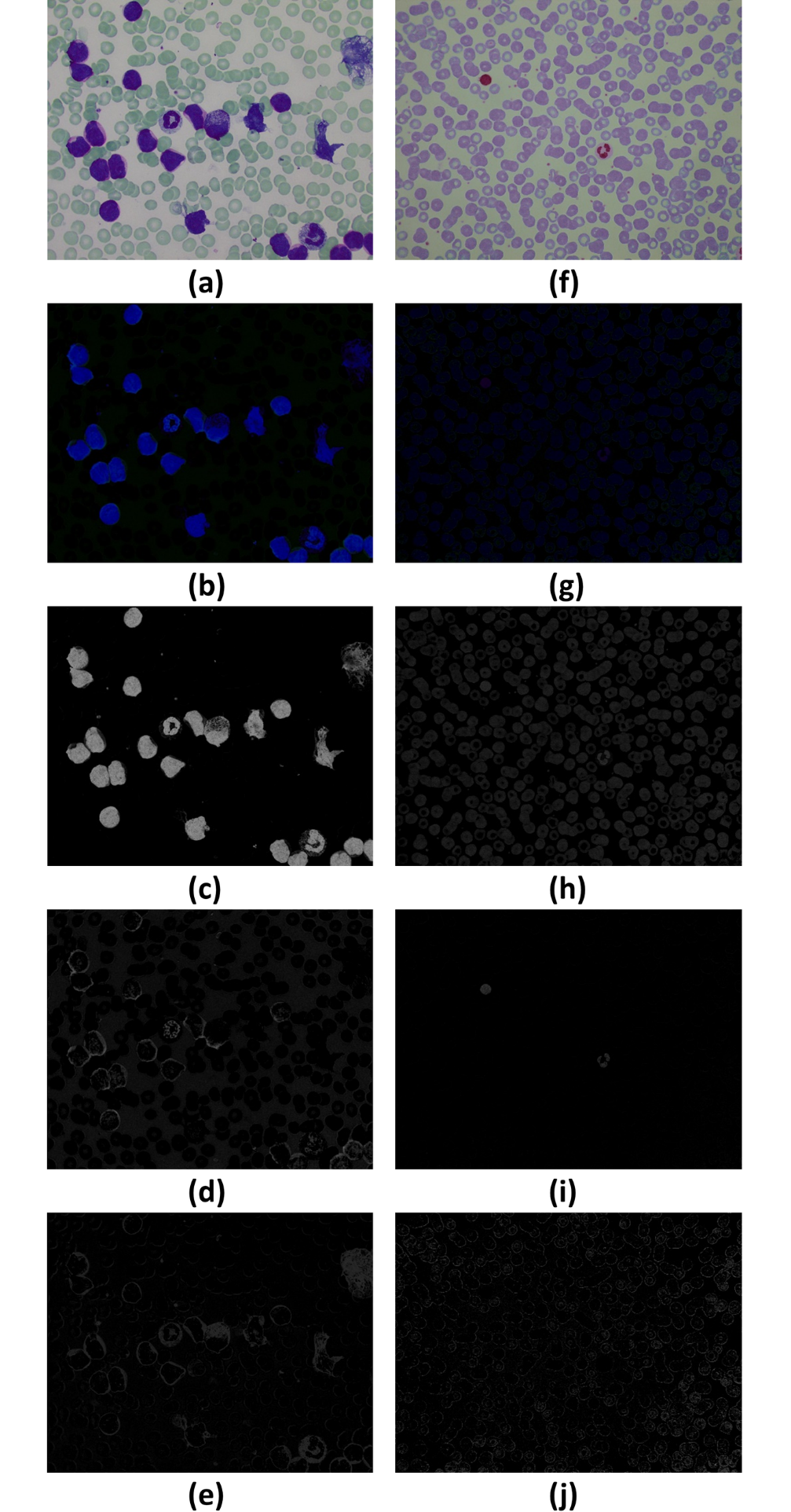


**Fig 5. Images obtained by processing the chromaticity in the HSV space of images of Fig 1. Images (a) and (f) were obtained by processing the chromaticity in the HSV space; images (b) and (g) were obtained after projecting images (a) and (f), respectively, to the eigenspace using RGB colors; images (c) and (h), (d) and (i), and (e) and (j) were obtained by projecting the colors to the first, second, and third PCs, respectively.**


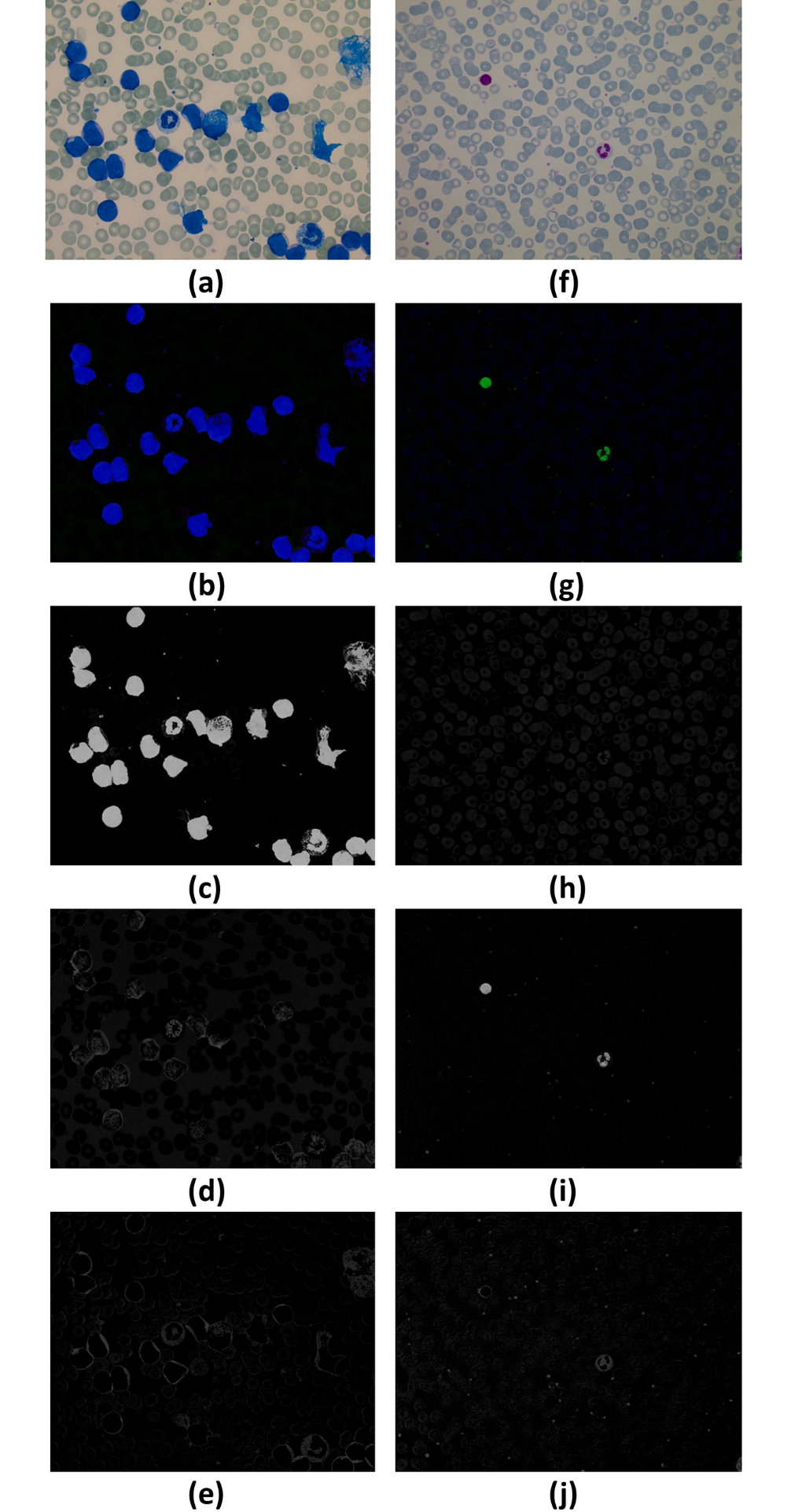


**Fig 6. Images obtained by processing the chromaticity in the L*a*b* space of images of Fig 1. Images (a) and (f) were obtained by processing the chromaticity in the L*a*b* space; images (b) and (g) were obtained after projecting the images (a) and (f), respectively, to the eigenspace using RGB colors; images (c) and (h), (d) and (i), and (e) and (j) were obtained by projecting the colors to the first, second, and third PCs, respectively.**


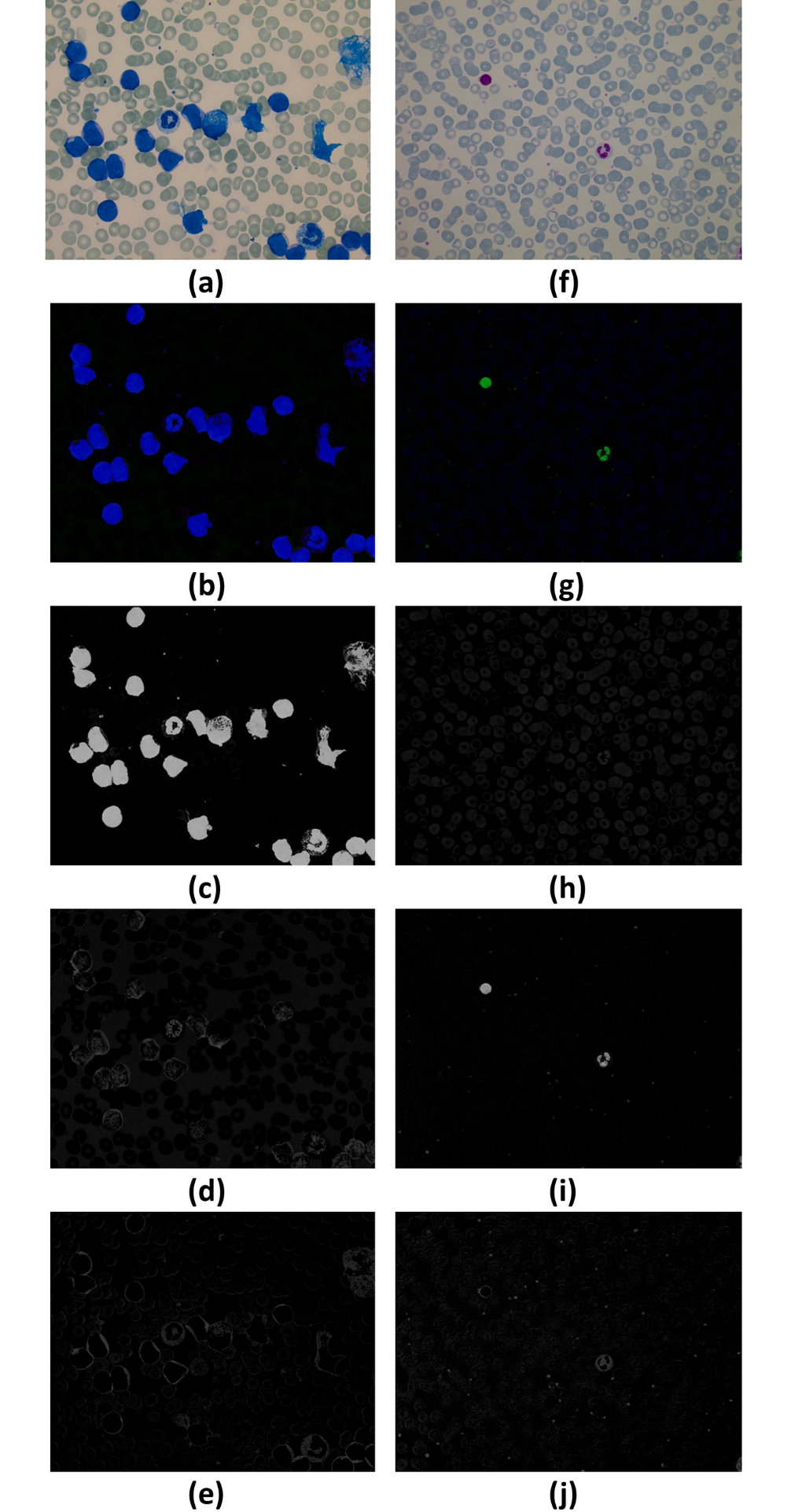


**Fig 7. Images used to illustrate our method. Images (a)–(d) and (e)–(h) extracted from the first 33 and last 75 images of the ALL-IDB, respectively**


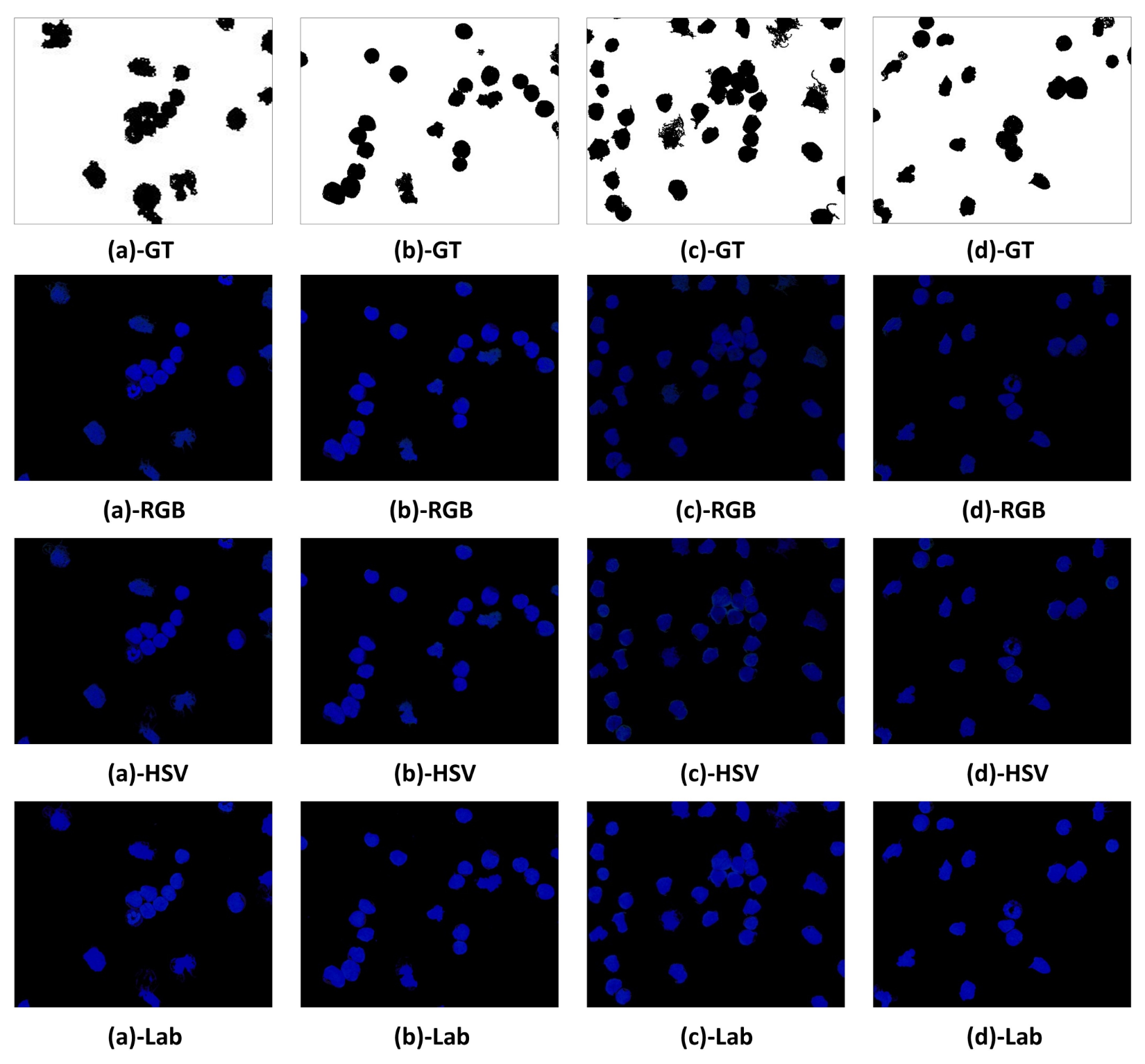


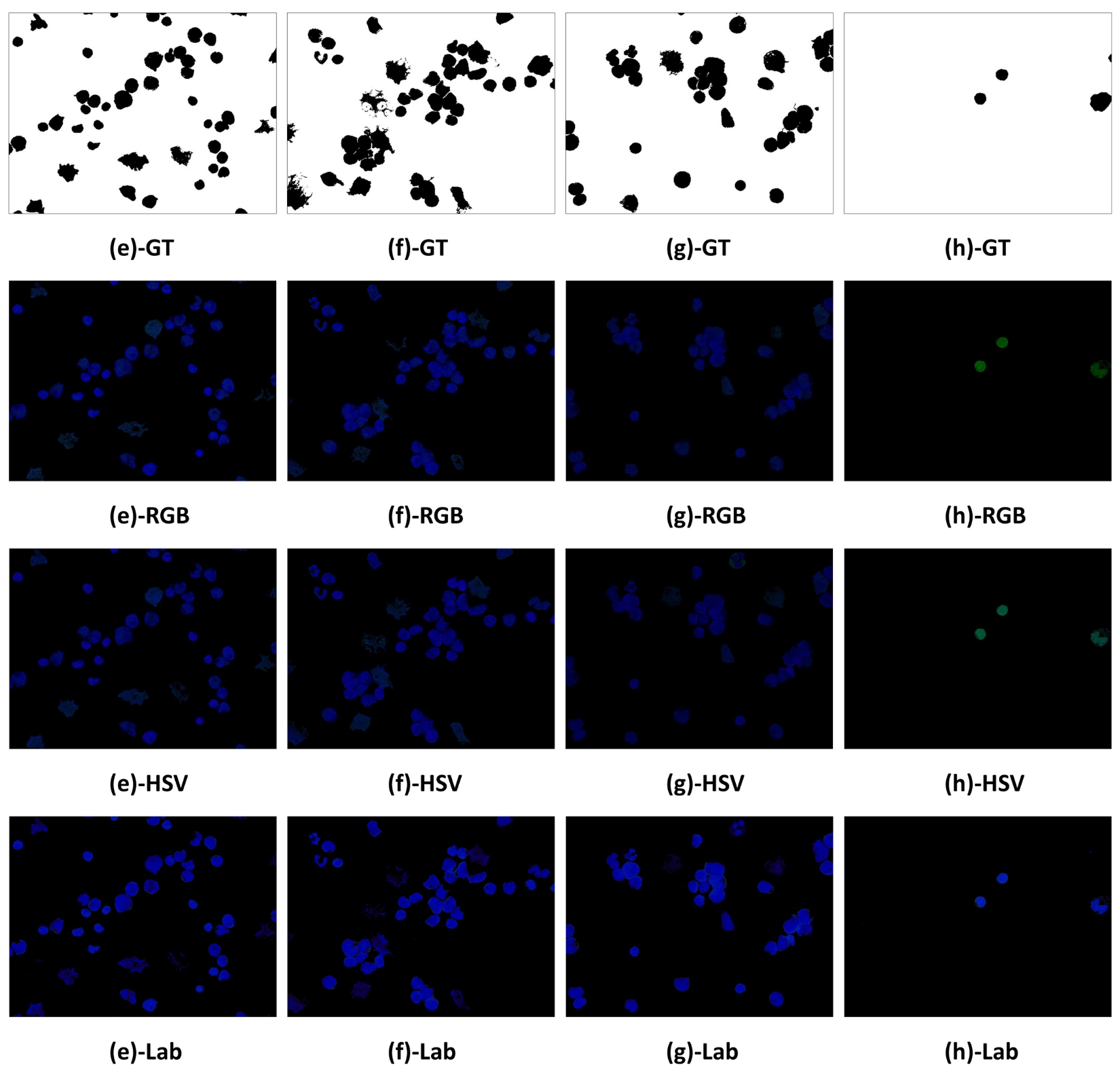


Fig 8. Images obtained after applying our approach to the images of Fig 7. Images {(a)-(d)}-GT ground truth, images {(a)-(d)}-RGB obtained using the RGB space, images {(a)-(d)}-HSV obtained using the HSV space, and images {(a)-(d)}-Lab obtained using the L*a*b* space.

**Fig 8 (continue). Images obtained after applying our approach to the images of Fig 7, processing the hue in the HSV space. Images {(e)-(h)}-GT ground truth, images {(e)-(h)}-RGB obtained using the RGB space, images {(e)-(h)}-HSV obtained using the HSV space, and images {(e)-(h)}-Lab obtained using the L*a*b* space.**
