# Supplementary material for: Systematic segmentation method based on PCA of image hue features for white blood cell counting
Source: PLoS One. 2021 Dec 31;16(12):e0261857. doi: 10.1371/journal.pone.0261857 (PMC8719728; doi:10.1371/journal.pone.0261857)
Supplement: Attachment — Submitted filename: PONE-D-21-23272_sl-review.pdf [file pone.0261857.s007.pdf]

PLOS ONE

Systematic segmentation method based on PCA of image hue features for white blood cell counting

--Manuscript Draft--

**Manuscript Number:**	PONE-D-21-23272
**Article Type:**	Research Article
**Full Title:**	Systematic segmentation method based on PCA of image hue features for white blood cell counting
**Short Title:**	Image-based white blood cell count
**Corresponding Author:**	Farid Garcia-Lamont, Ph.D.<br>Universidad Autonoma del Estado de Mexico<br>Texcoco, Estado de Mexico MEXICO
**Keywords:**	white blood cells; WBC counting automation; chromaticity as a feature; color spaces; image segmentation
**Abstract:**	Leukocyte (white blood cell, WBC) count is an essential factor that physicians use to diagnose infections and provide adequate treatment. Currently, WBC count is determined manually or semi-automatically, which often leads to miscounting. In this paper, we propose an automated method that uses a bioinspired segmentation mimicking the human perception of color. It is based on the claim that a person can locate WBCs in a blood smear image via the high chromatic contrast. By applying principal component analysis over RGB, HSV, and L*a*b* spaces, pixels of leukocytes present high chromatic variance, which is in high contrast with the average hue of the other blood smear elements. Second, chromaticity is processed as a feature, without separating hue components in a trial-and-error approach, which is used in most of the current automation. As a result of this systematic method, WBC recognition is computationally efficient, overlapping WBCs are separated, and the final count is more precise. In experiments with the ALL-IDB benchmark, the performance of the proposed segmentation was assessed by comparing the WBC from the processed images with the ground truth. Compared with previous methods, the proposed method achieved similar results in sensitivity and precision and approximately 0.2% higher specificity and 0.3% higher accuracy.
**Order of Authors:**	Farid Garcia-Lamont, Ph.D.
	Matias Alvarado
	Jair Cervantes
**Additional Information:**	
**Question**	**Response**
**Financial Disclosure**<br><br>Enter a financial disclosure statement that describes the sources of funding for the work included in this submission. Review the submission guidelines for detailed requirements. View published research articles from *PLOS ONE* for specific examples.<br><br>This statement is required for submission and **will appear in the published article** if the submission is accepted. Please make sure it is accurate.	Initials: M.A. (Matias Alvarado)<br><br>Grant number:A1-S-20037<br><br>Funder name: National Council of Research (CONACyT in Spanish abbreviation)<br><br>The funders had no role in study design, data collection and analysis, decision to publish, or preparation of the manuscript.

Powered by Editorial Manager® and ProduXion Manager® from Aries Systems Corporation

**Unfunded studies**<br>Enter: *The author(s) received no specific funding for this work.*<br><br>**Funded studies**<br>Enter a statement with the following details:<br>• Initials of the authors who received each award<br>• Grant numbers awarded to each author<br>• The full name of each funder<br>• URL of each funder website<br>• Did the sponsors or funders play any role in the study design, data collection and analysis, decision to publish, or preparation of the manuscript?<br>• **NO** - Include this sentence at the end of your statement: *The funders had no role in study design, data collection and analysis, decision to publish, or preparation of the manuscript.*<br>• **YES** - Specify the role(s) played.<br><br>* typeset	
**Competing Interests**<br><br>Use the instructions below to enter a competing interest statement for this submission. On behalf of all authors, disclose any competing interests that could be perceived to bias this work—acknowledging all financial support and any other relevant financial or non-financial competing interests.<br><br>This statement is required for submission and **will appear in the published article** if the submission is accepted. Please make sure it is accurate and that any funding sources listed in your Funding Information later in the submission form are also declared in your Financial Disclosure statement.<br><br>View published research articles from *PLOS ONE* for specific examples.	The authors have declared that no competing interests exist.

Powered by Editorial Manager® and ProduXion Manager® from Aries Systems Corporation

**NO authors have competing interests**<br><br>Enter: *The authors have declared that no competing interests exist.*<br><br>**Authors with competing interests**<br><br>Enter competing interest details beginning with this statement:<br><br>*I have read the journal's policy and the authors of this manuscript have the following competing interests: [insert competing interests here]*<br><br>* typeset	
**Ethics Statement**<br><br>Enter an ethics statement for this submission. This statement is required if the study involved:<br><br>• Human participants<br>• Human specimens or tissue<br>• Vertebrate animals or cephalopods<br>• Vertebrate embryos or tissues<br>• Field research<br><br>Write "N/A" if the submission does not require an ethics statement.<br><br>General guidance is provided below. Consult the submission guidelines for detailed instructions. **Make sure that all information entered here is included in the Methods section of the manuscript.**	This paper does not contain any studies with animals or humans performed by any of the authors.

**Format for specific study types**<br><br>**Human Subject Research (involving human participants and/or tissue)**<br>• Give the name of the institutional review board or ethics committee that approved the study<br>• Include the approval number and/or a statement indicating approval of this research<br>• Indicate the form of consent obtained (written/oral) or the reason that consent was not obtained (e.g. the data were analyzed anonymously)<br><br>**Animal Research (involving vertebrate animals, embryos or tissues)**<br>• Provide the name of the Institutional Animal Care and Use Committee (IACUC) or other relevant ethics board that reviewed the study protocol, and indicate whether they approved this research or granted a formal waiver of ethical approval<br>• Include an approval number if one was obtained<br>• If the study involved *non-human primates*, add *additional details* about animal welfare and steps taken to ameliorate suffering<br>• If anesthesia, euthanasia, or any kind of animal sacrifice is part of the study, include briefly which substances and/or methods were applied<br><br>**Field Research**<br><br>Include the following details if this study involves the collection of plant, animal, or other materials from a natural setting:<br>• Field permit number<br>• Name of the institution or relevant body that granted permission	
**Data Availability**<br><br>Authors are required to make all data underlying the findings described fully available, without restriction, and from the time of publication. PLOS allows rare exceptions to address legal and ethical concerns. See the PLOS Data Policy and FAQ for detailed information.	Yes - all data are fully available without restriction

A Data Availability Statement describing where the data can be found is required at submission. Your answers to this question constitute the Data Availability Statement and **will be published in the article**, if accepted.<br><br>**Important:** Stating 'data available on request from the author' is not sufficient. If your data are only available upon request, select 'No' for the first question and explain your exceptional situation in the text box.<br><br>Do the authors confirm that all data underlying the findings described in their manuscript are fully available without restriction?	
**Describe where the data may be found in full sentences. If you are copying our sample text, replace any instances of XXX with the appropriate details.**<br><br>• If the data are **held or will be held in a public repository**, include URLs, accession numbers or DOIs. If this information will only be available after acceptance, indicate this by ticking the box below. For example: *All XXX files are available from the XXX database (accession number(s) XXX, XXX.).*<br>• If the data are all contained **within the manuscript and/or Supporting Information files**, enter the following: *All relevant data are within the manuscript and its Supporting Information files.*<br>• If neither of these applies but you are able to provide **details of access elsewhere**, with or without limitations, please do so. For example:<br><br>*Data cannot be shared publicly because of [XXX]. Data are available from the XXX Institutional Data Access / Ethics Committee (contact via XXX) for researchers who meet the criteria for access to confidential data.*<br><br>*The data underlying the results presented in the study are available from (include the name of the third party*	All relevant data are within the manuscript and its Supporting Information files.

and contact information or URL).<br>• This text is appropriate if the data are owned by a third party and authors do not have permission to share the data.<br><br>* typeset	
Additional data availability information:	

Powered by Editorial Manager® and ProduXion Manager® from Aries Systems Corporation

Manuscript

Click here to access/download;Manuscript;Without_Tracks_PCA_GarciaF_Alv

Systematic segmentation method based on PCA of image hue features for white blood cell counting

Short title: Image-based white blood cell count

Farid García-Lamont[1*], Matías Alvarado[2], Jair Cervantes[1]

[1]Universidad Autónoma del Estado de México, Centro Universitario UAEM Texcoco, Texcoco-Estado de México, México

[2]Centro de Investigación y de Estudios Avanzados del IPN, Departamento de Computación, CDMX-México

*Corresponding author

E-mail: fgarcial@uaemex.mx

Abstract

Leukocyte (white blood cell, WBC) count is an essential factor that physicians use to diagnose infections and provide adequate treatment. Currently, WBC count is determined manually or semi-automatically, which often leads to miscounting. In this paper, we propose an automated method that uses a bioinspired segmentation mimicking the human perception of color. It is based on the claim that a person can locate WBCs in a blood smear image via the high chromatic contrast. By applying principal component analysis over RGB, HSV, and L*a*b* spaces, pixels of leukocytes present high chromatic variance, which is in high contrast with the average hue of the other blood smear elements. Second, chromaticity is processed as a feature, without separating hue components in a *trial-and-error* approach, which is used in most of the current automation. As a result of this systematic method, WBC recognition is computationally efficient, overlapping WBCs are separated, and the final count is more precise. In experiments with the ALL-IDB benchmark, the performance of the proposed segmentation was assessed by comparing the WBC from the processed images with the ground truth. Compared with previous methods, the proposed method achieved similar results in sensitivity and precision and approximately 0.2% higher specificity and 0.3% higher accuracy.

Keywords: White blood cells, WBC counting automation, chromaticity as a feature, color spaces, image segmentation

Introduction

White blood cell (WBC) count is an important factor that physicians employ to diagnose illnesses and to assess and identify adequate treatment [1-5]. The WBC counting process is performed as follows: a blood smear (BS) sample is extracted from the patient and the sample is stained so that the WBCs become visible; a hematologist then counts the WBCs in the sample using an optic microscope.

Often, the WBC count is not automated, and the process is slow. In such cases, the counting precision depends on the fatigue level of the hematologist after analyzing several samples, which could lead to involuntary miscounting. With an automated vision system implemented to perform this

task, more samples could be analyzed accurately and quickly. Moreover, automation of WBC count would enable physicians to focus on providing a correct diagnosis, which could prevent the expansion of the infection.

In artificial vision systems addressing WBC counting [6-14], an important stage in the process is the segmentation because precise counting depends on isolating WBCs from other elements of the BS, and separating overlapping WBCs.

The contribution of related studies is primarily in the development of WBC segmentation techniques in BS images [3, 7, 9, 10, 12-25]; within these and other techniques, color data are employed [7, 13, 14, 17-19, 21, 23-25]. However, through these methods, color is not processed as a feature because the color components, as if they were *intensity* channels, are decoupled and processed separately. Furthermore, the color components or channels are intuitively operated within a type of *trial-and-error* approach. We attempt to overcome this weakness and propose a systematic operational method.

Our segmentation proposal is bioinspired and mimics the human perception of color. We claim that any person can differentiate WBCs from other elements of the BS based on their color characteristics. Considering the BS images in Fig 1, although captured under different staining, scale, and illumination conditions, it is easy for a human to locate the WBCs within the image because the color of the WBCs contrasts with the colors of the other elements of the BS. Hence, we propose segmenting the WBCs by selecting pixels with high chromatic variance when compared with the average color.

Fig 1. Blood smear images captured with different cameras and under different staining, scale, and illumination conditions.

We maximize the chromatic variance of the pixels by projecting the hue of the pixels to the eigenspace created from the principal components (PCs) computed with the hue data of the given image. Therefore, the data are uncorrelated, and they are oriented to obtain the highest variance. This results in an improved precision of segmentation, as we show below.

Although the red-green-blue (RGB) space is often employed to represent colors, it is not adequate for color processing because the color differences cannot be computed using the Euclidean distance [26]. Thus, we performed experiments using the L*a*b* and hue-saturation-value (HSV) color spaces to process the chromaticity. These color spaces are adequate for color processing, and the representation resembles the human perception of color because the chromaticity and intensity are decoupled [26]. In addition, we propose to separate overlapping WBCs: the intensity gradient and chromatic difference of the pixels are employed for border detection; thus, the accuracy of the WBC count is improved as well.

~~In summary~~, the contribution of this study is the systematic operational method for WBC counting. The WBCs are segmented by selecting pixels with high chromatic variance via PC analysis to maximize the variance, and a technique for separating overlapping WBCs is proposed.

The remainder of the paper is organized as follows: the segmentation and counting methodology is introduced in Section 0; in Section 0 the experiments and results are presented; the results are discussed in Section 0; and the paper closes with conclusions presented in Section 0.

Methods and proposal

The methodology consists of the following steps. First, the RGB image is mapped to another color space. In this study, we performed experiments using the HSV and L*a*b* spaces. The hue component is extracted and projected to the eigenspace built with the PCs computed with the hue

data; these projections are mapped back to the RGB space. These "new" RGB color vectors are projected to the eigenspace generated by the PCs obtained with these RGB color vectors (Fig 2). Thereby, the WBCs are enhanced. After the WBCs are segmented, overlapping WBCs are separated. Then, the resulting image is binarized, and connected component labeling is employed to count the WBCs.

Fig 2. Flow chart of proposed methodology. ϕ_k is the RGB color vector of the kth pixel; φ_k is the color vector obtained after mapping the vector ϕ_k to a different color space; the vector ψ_k contains the hue components of the vector φ_k; the vector ψ_k is mapped to the eigenspace obtaining the vector ψ_k^*; the hue components of the vector φ_k are substituted by the vector ψ_k^* obtaining the vector φ_k^*; the vector φ_k^* is mapped to the RGB space, obtaining the vector ϕ_k^*; the vector ϕ_k^* is normalized, obtaining the vector $\hat{\phi}_k^*$; the vector $\hat{\phi}_k^*$ is projected to the eigenspace, obtaining the vectors ω_k.

PC analysis takes a dataset and finds a new orthonormal basis with its axes ordered, called PCs. This new basis is rotated in such a way that the first axis is oriented along the direction in which the data have their highest variance. Consequently, these axes have associated decreasing "indices" corresponding to the variance of the dataset when projected on the axes. PCs are ordered according to their corresponding eigenvalues or variances [27]. Computing PCs involves the following mathematical operations:

Let a dataset be represented as a set of column vectors $\{\mathbf{x}_1, \ldots, \mathbf{x}_m\} \subset \mathbb{R}^n$, and the mean of the vectors is computed as

$$\mu_{\mathbf{x}} = \frac{1}{m}\sum_{k=1}^{m} \mathbf{x}_k. \quad (1)$$

The covariance matrix is obtained:

$$\Omega = \frac{1}{m} \Phi \Phi^T \quad (2)$$

Here, $\Phi = [\mathbf{x}_1 - \mu_{\mathbf{x}}, \dots, \mathbf{x}_m - \mu_{\mathbf{x}}]$. The eigenvalues and eigenvectors of the covariance matrix are computed. The eigenvectors are ordered according to their associated eigenvalues, from the smallest to the largest, and placed as columns of the matrix $\Lambda = [\mathbf{v}_1, \dots, \mathbf{v}_n]$, where $\lambda_1 < \lambda_2 < \cdots < \lambda_n$.

The vectors of the set $\{\mathbf{x}_1, \dots, \mathbf{x}_m\}$ are projected to the eigenspace as

$$\mathbf{y}_k = \Lambda^T (\mathbf{x}_k - \mu_{\mathbf{x}}), k = 1, \dots, m. \quad (3)$$

The column vectors $\mathbf{y}_k$ are the respective projections to the eigenspace of the vectors of the original dataset.

Segmentation approach

The segmentation ~~proposal~~ consists of the following steps:

1. Let $\{\phi_{(i,j)} | \forall (i,j) \in T\} \subset \mathbb{R}^3$ be the set of RGB color vectors of the given image, where $T = \{1, \dots, n\} \times \{1, \dots, m\}$, and n and m are the numbers of rows and columns of the input image, respectively. The color vectors are mapped to a different color space, obtaining the set of vectors $\{\varphi_{(i,j)} | \forall (i,j) \in T\} \subset \mathbb{R}^3$.
2. The hue component is extracted and represented by the vectors of the set $\{\psi_{(i,j)} | \forall (i,j) \in T\} \subset \mathbb{R}^2$. A new eigenspace is built with these vectors by computing the PCs. The vectors $\psi_{(i,j)}$ are projected to this eigenspace, obtaining the set of vectors $\{\psi^*_{(i,j)} | \forall (i,j) \in T\}$.
3. The hue component of the vectors $\varphi_{(i,j)}$ is substituted by the respective vectors $\psi^*_{(i,j)}$ to obtain the set of vectors $\{\varphi^*_{(i,j)} | \forall (i,j) \in T\}$.
4. The vectors φ^*_m are mapped to the RGB space to obtain the set of vectors $\{\phi^*_{(i,j)} | \forall (i,j) \in T\}$, which are normalized with $\hat{\phi}^*_{(i,j)} = \phi^*_{(i,j)} / \|\phi^*_{(i,j)}\|$ and placed in the set $\{\hat{\phi}^*_{(i,j)} | \forall (i,j) \in T\}$.

The PCs $\{\mathbf{v}_1, \mathbf{v}_2, \mathbf{v}_3\} \subset \mathbb{R}^3$ and the respective eigenvalues λ_1, λ_2, and λ_3 are computed from the vectors $\hat{\phi}^*_{(i,j)}$, where $\lambda_1 < \lambda_2 < \lambda_3$.

The vectors $\hat{\phi}^*_{(i,j)}$ are projected to the eigenspace:

$$\omega_{(i,j)} = \mathbf{v}_p^T\left(\hat{\phi}^*_{(i,j)} - \mu_{\hat{\phi}^*}\right), \forall (i,j) \in T, \quad (4)$$

where, $\mu_{\hat{\phi}^*}$ is computed using Eq. (1). Experimentally, we found that depending on the color space used and the proportion of the accumulative variance contained by the largest eigenvalue, the segmentation of the WBCs is obtained by projecting the vectors $\hat{\phi}^*_{(i,j)}$ with the first or the second PC; that is,

$$\mathbf{v}_p = \begin{cases} \mathbf{v}_3, & \dfrac{\lambda_3}{\sum_{i=1}^{3} \lambda_i} \geq \delta_\lambda \\ \mathbf{v}_2, & \text{otherwise.} \end{cases} \quad (5)$$

Here, $\delta_\lambda = 0.9$ when the L*a*b* space is employed and $\delta_\lambda = 0.75$ when the hue is processed in the RGB and HSV spaces.

Hue characterization

In Step 2 of the segmentation approach, the chromaticity is characterized as follows. In the L*a*b* space, components a and b are used to characterize the hue with

$$\psi_k = [a_k, b_k]^T. \quad (6)$$

Once the vectors are projected to the eigenspace, $\psi^*_k = [a^*_k, b^*_k]^T$, they are substituted in the vectors φ^*_k, which are then mapped to the RGB space.

In the HSV space, component h is employed, and the hue is characterized by

$$\psi_k = [\cos h_k, \sin h_k]^T. \quad (7)$$

Once the vectors are projected to the eigenspace, $\psi_k^* = [x_k, y_k]^T$, the orientation of the vectors is computed in terms of the HSV space with

$$h_k^* = \begin{cases} \tan^{-1}\left(\frac{y_k}{x_k}\right), & x_k > 0, y_k > 0 \\ \pi - \tan^{-1}\left(\frac{y_k}{|x_k|}\right), & x_k < 0, y_k > 0 \\ \pi + \tan^{-1}\left(\frac{y_k}{x_k}\right), & x_k < 0, y_k < 0 \\ 2\pi - \tan^{-1}\left(\frac{|y_k|}{x_k}\right), & x_k > 0, y_k < 0 \\ \frac{\pi}{2}, & x_k = 0, y_k > 0 \\ \frac{3}{2}\pi, & x_k = 0, y_k < 0. \end{cases} \quad (8)$$

The value of h_k^* is substituted in the vectors φ_k^*, then they are mapped to the RGB space. Reference [26] shows the mathematical operations to map the colors between the RGB, L*a*b*, and HSV spaces.

WBC counting

The image obtained with vector projections $\omega_{(i,j)}$, Eq. (4), is binarized as follows.

$$B_{(i,j)} = \begin{cases} 1, & \left|\omega_{(i,j)}\right| \geq \delta_\omega \\ 0, & \text{otherwise.} \end{cases} \quad (9)$$

The WBCs are counted using the connected component labeling to the binary image $B_{(i,j)}$.

Before the WBCs are counted, the overlapping WBCs are separated by computing their borders using the gradient method. We include the chromatic difference between pixels by obtaining the orientation difference between the color vectors represented in the RGB space. A pixel is set to zero if detected as a border:

$$B_{(i,j)} = \begin{cases} 1, & \left\|\nabla\omega_{(i,j)}\right\| > \delta_I, \theta_{(i,j)}^x > \delta_\theta, \theta_{(i,j)}^y > \delta_\theta \\ 0, & \text{otherwise,} \end{cases} \quad (10)$$

where $\nabla\omega_{(i,j)}$ is the intensity gradient of the channel created with the projections $\omega_{(i,j)}$ using the Prewitt masks; $\theta^x_{(i,j)}$ and $\theta^y_{(i,j)}$ are the orientation difference of the color vectors in the vertical and horizontal directions, respectively, computed as follows:

$$\theta^x_{(i,j)} = \cos^{-1}\left(\hat{\phi}^*_{(i,j)} \cdot \hat{\phi}^*_{(i+1,j)}\right), \tag{11}$$

$$\theta^y_{(i,j)} = \cos^{-1}\left(\hat{\phi}^*_{(i,j)} \cdot \hat{\phi}^*_{(i,j+1)}\right). \tag{12}$$

The separation approach is only applied to large objects; however, before the overlapping WBCs are separated, small objects are deleted.

Experiments and results

We employed the well-known Acute Lymphoblastic Leukaemia Image Database* (ALL-IDB), which is a benchmark database used to test algorithms for counting, cancer detection, and classification of WBCs. The ALL-IDB contains 108 color images of BS samples: a set of 33 images of 1368 × 1712-pixel size and a set of 75 images of 1944 × 2592-pixel size. Images (a) and (b) of Fig 1 belong to the first and second sets, respectively. Most of the images of the ALL-IDB contain more than one WBC and several of the WBCs overlap.

Quantitative evaluation of segmented images

We conducted a pixel-level segmentation evaluation by comparing the segmented images with their corresponding ground truths†. The quantitative evaluation was computed using the metrics: accuracy (*Ac*), specificity (*Sp*), precision (*Pr*), sensitivity (*Se*), f-score (*f*), and kappa index (*K*). See Equations (13)-(19).

* https://homes.di.unimi.it/scotti/all/
† https://drive.google.com/drive/folders/1F7kZ1SRWUD9R6aHLMkj3wsjcHnvlGuwP?usp=sharing

$$Ac = \frac{TP + TN}{TP + TN + FP + FN} \quad (13)$$

$$Sp = \frac{TN}{TN + FP} \quad (14)$$

$$Pr = \frac{TP}{TP + FP} \quad (15)$$

$$Se = \frac{TP}{TP + FN} \quad (16)$$

$$f = \frac{2TP}{2TP + FP + FN} \quad (17)$$

$$K = \frac{Ac - t}{1 - t}, \quad (18)$$

where

$$t = \frac{(TP + FN) \times (TP + FP) + (TN + FN) \times (TN + FP)}{(TP + TN + FP + FN)^2}. \quad (19)$$

The accuracy level in relation to K is classified as follows [28]: poor ($K \leq 0.2$), reasonable ($0.2 < K \leq 0.4$), good ($0.4 < K \leq 0.6$), very good ($0.6 < K \leq 0.8$), and excellent ($K > 0.8$).

Table 1 summarizes the pixel classification of true positives (TP), false positives (FP), true negatives (TN), and false negatives (FN).

Table 1. Definition of pixels classified as true positives (TP), false positives (FP), true negatives (TN), and false negatives (FN)

	**Ground truth**	**Classified as**
**TP**	WBC	WBC
**FP**	Background	WBC
**TN**	Background	Background
**FN**	WBC	Background

Fig 3 shows the corresponding ground truth of the images in Fig 1.

Fig 3. Ground truth of the images of Fig 1.

Table 2 shows the results obtained using Eqs. (13)-(19), where the threshold value in Eq. (9) is $\delta_\omega = 37$. It is easy to appreciate that the highest performance is obtained by processing the chromaticity in the L*a*b* space: $K = 0.8589$, and the accuracy level, in relation to *K,* is classified as excellent: $K > 0.8$.

Table 2. Results obtained by comparing the resulting images with the ground truth. The highest values are italicized. The chromaticity is processed in the RGB, L*a*b*, and HSV spaces.

Method	Sensitivity	Specificity	Precision	Accuracy	f-score	*K*
RGB	0.6665	*0.9999*	*0.9916*	0.9895	0.7972	0.7920
HSV	0.6783	0.9998	0.9881	0.9897	0.8044	0.7993
L*a*b*	*0.7671*	0.9997	0.9857	*0.9924*	*0.8628*	*0.8589*

Fig 4 shows the segmented images obtained by processing the hue in the RGB space of the images in Fig 1.

Fig 4. Images obtained by processing the chromaticity in the RGB space of images of Fig 1. Images (a) and (e) were obtained after projecting the colors onto the eigenspace, and images (b) and (f), (c) and (g), and (d) and (h) resulted from projecting the colors with the first, second, and third PCs, respectively.

Fig 5 shows the images obtained by processing the chromaticity in the HSV space of images of Fig 1**.**

Fig 5. Images obtained by processing the chromaticity in the HSV space of images of Fig 1. Images (a) and (f) were obtained by processing the chromaticity in the HSV space; images (b) and (g) were obtained after projecting images (a) and (f),

respectively, to the eigenspace using RGB colors; images (c) and (h), (d) and (i), and (e) and (j) were obtained by projecting the colors to the first, second, and third PCs, respectively.

Fig 6 shows the images obtained by processing the chromaticity in the L*a*b* space of images of Fig 1**.**

Fig 6. Images obtained by processing the chromaticity in the L*a*b* space of images of Fig 1. Images (a) and (f) were obtained by processing the chromaticity in the L*a*b* space; images (b) and (g) were obtained after projecting the images (a) and (f), respectively, to the eigenspace using RGB colors; images (c) and (h), (d) and (i), and (e) and (j) were obtained by projecting the colors to the first, second, and third PCs, respectively.

Images (a)–(d) and (e)–(h) of Fig 7 are extracted from the first 33 and last 75 images of the ALL-IDB, respectively. Fig 8, Fig 9 and Fig 10 show the resulting images by applying our approach to the images in Fig 7, where the hue is processed in the RGB, HSV, and L*a*b* spaces, respectively.

Fig 7. Images used to illustrate our method. Images (a)–(d) and (e)–(h) extracted from the first 33 and last 75 images of the ALL-IDB, respectively.

Fig 8. Images obtained after applying our approach to the images of Fig 7, processing the hue in the RGB space.

Fig 9. Images obtained after applying our approach to the images of Fig 7, processing the hue in the HSV space.

Fig 10. Images obtained after applying our approach to the images of Fig 7, processing the hue in the L*a*b* space.

WBC counting

After the segmented image was binarized, as explained in Section 0, connected component labeling was applied to count the WBCs. The counting precision was computed using Eq. (20):

$$Cp = \frac{N}{M} \times 100, \qquad (20)$$

where N is the number of correctly detected WBCs, and M is the total number of detected WBCs. Table 3 shows the counting results, with the threshold values of Eq. (10) set to $\delta_I = 0.2$ and $\delta_\theta = 2.5°$.

Table 3. Counting precision obtained using the RGB, HSV, and L*a*b* spaces and separating overlapping WBCs.

Color space	Counting precision
RGB	86.14%
HSV	64.78%
L*a*b*	95.12%

For the first 33 images and last 75 images, objects smaller than 3500 and 5000 pixels, respectively, were deleted. Analogously, the separation approach was applied to objects larger than 13,000 and 21,000 pixels for the first 33 images and the last 75 images, respectively.

Discussion

Segmentation and counting

References [7], [10], [25], and [29] are the only previous works we found that reported quantitative evaluation of their resulting images regarding the ALL-IDB ground truth. Table 4 shows the results reported in these works and ours.

Table 4. Quantitative evaluation comparison of segmented images regarding the ALL-IDB ground truth, using different methods. The highest values are italicized.

Reference	Sensitivity	Specificity	Precision	Accuracy	f-score
Safuan et al. [7]	*0.9910*	0.9687	-	0.9887	-
Di Ruberto et al. [10]	0.9845	0.9756	0.7045	0.9761	0.8213
Sudha and Geetha [25]	0.9805	-	*0.9932*	-	*0.9867*
Shahzad et al. [29]	-	-	-	0.9334	0.3309
Our proposal (L*a*b*)	0.7671	*0.9997*	0.9857	*0.9924*	0.8628

The results presented in [7] and [10] were obtained by processing only the first 30 and 33 images of the ALL-IDB, respectively. In reference [25], the hue features were not employed for segmentation, and only the S channel was used after mapping the given RGB image to the HSV space. Shahzad et al. [29] used a deep convolutional encoder-decoder network for semantic segmentation, which was fed with grayscale images; thus, they did not use color features.

The value of 1) *specificity* refers to the segmentation quality of the background in the images; 2) *precision* refers to the quality of the segmented images in that the pixels of the background are not classified as part of WBC; 3) *sensitivity* refers to the quality of the segmented images, in that the pixels of the WBC are not classified as part of the background; and 4) *accuracy* refers to the quality of the classified pixels, both the WBC and background pixels. Our proposed method outperforms the previous methods in specificity and accuracy (Table 4), and thus improves the quality of the segmentation regarding the background and the entire WBC image content.

In state-of-the-art image segmentation, the algorithms are usually validated with regard to the ground truth, using the probabilistic random index (PRI), variation of information (VOI), and global consistency error (GCE) [26]. Studies addressing segmentation of WBCs validate the algorithms by

evaluating the classification at the pixel level of the segmented images, but with this approach, the object-based evaluation of segmentation cannot be performed. With the PRI, VOI, and GCE metrics, it is possible to conduct an object-based evaluation of the segmented images.

Let I and S be the ground truth and segmentation provided by the algorithm, respectively. PRI measures the similarity between the two data clusters as follows:

$$PRI(S,I) = \frac{2}{n(n-1)} \sum_{i,j,i<j} p_{i,j}^{c_{i,j}} \left(1 - p_{i,j}\right)^{c_{i,j}}, \tag{21}$$

where n is the number of pixels; $c_{i,j}$ is a Boolean function: $c_{i,j} = 1$ if $L_i^I = L_j^S$ and $c_{i,j} = 0$ otherwise; L_i^I is the label of pixel x_i in the ground truth; L_j^S is the label of pixel x_j in the segmented image; and $p_{i,j}$ is the expected value of the Bernoulli distribution for the pixel pair.

The VOI index measures the sum of the loss and gain of information between the two clusters belonging to the lattice of possible partitions as follows:

$$VOI(S,I) = H(S) + H(I) - 2F(S,I), \tag{22}$$

where $H = -\sum_{i=1}^{c} (n_i/n) \log(n_i/n)$, n_i is the number of points belonging to the *i*th cluster, and c is the number of clusters. F is the mutual information between the two clusters, defined as

$$F(S,I) = \sum_{i=1}^{c_S} \sum_{j=1}^{c_I} \frac{n_{i,j}}{n} \log \frac{n_i n_j}{n^2}, \tag{23}$$

where $n_{i,j}$ is the number of points in the intersection of cluster i of S and j of I, and c_S and c_I are the numbers of clusters of S and I, respectively.

The GCE computes the extent a segmented image is viewed as a refinement of the other. A measure of the error at each pixel x_i can be written as

$$GCE(S,I,x_i) = \frac{1}{n} \min\left(\sum_{i=1}^{n} C(S,I,x_i), \sum_{i=1}^{n} C(I,S,x_i)\right), \tag{24}$$

where $C(S, I, x_i) = |R(S, x_i) \setminus R(I, x_i)| \div |R(S, x_i)|$, $|\cdot|$ is the cardinality, $\setminus$ is the set difference, and $R(S, x_i)$ is the set of pixels belonging to the region in segmentation S containing pixel x_i.

The ranges of the PRI, VOI, and GCE metrics are $[0,1]$, $[0, \infty)$, and $[0,1]$, respectively. The higher the value of PRI, the better the segmentation, and the lower the value of VOI and GCE, the better the segmentation with respect to the ground truth. Table 5 shows the average PRI, VOI, and GCE values obtained by evaluating the segmented images.

Table 5. Average PRI, VOI, and GCE values of the resulting images. The highest values are italicized.

Color space	PRI	VOI	GCE
RGB	0.8674	0.3398	*0.0147*
HSV	0.6967	0.7205	0.0301
L*a*b*	*0.8891*	*0.3091*	0.0156

The results obtained with the PRI, VOI, and GCE metrics cannot be compared with related works addressing WBC segmentation because these metrics have not been used to evaluate the resulting images in these works. However, given the results shown in Table 5 it is easy to appreciate that the object-based evaluation similitude between the segmented images and the ground truth is high. The best results for PRI and VOI were obtained with the L*a*b* space, while the best value for GCE was obtained with the RGB space, but the respective value obtained with the L*a*b* space is very close.

It is important to note that most of the ground truth WBCs are segmented with the cytoplasm, whereas with our approach, the WBCs are segmented without the cytoplasm, only the nucleus. Therefore, the cytoplasm pixels were classified as the background; hence, sensitivity achieved the lowest value. Nevertheless, the WBC count was not affected since WBCs were detected.

In Table 6 we compare the counting precision obtained with our approach with previous works that use the ALL-IDB.

Table 6. Counting precision comparison of different works. The highest values italicized.

References	Counting precision
Safuan et al. [7]	96.92%
Di Ruberto et al. [8]	*99.20%*
Di Ruberto et al. [10]	97.61%
Alomari et al. [11]	98.40%
Our proposal (L*a*b*)	95.12%

According to Table 6, the performance of our proposal is close to the results reported in the cited works. The results obtained in references [10] and [7] were obtained by processing only the first 33 and 30 images of the ALL-IDB, respectively. We obtained 94.08% and 94.24% precision for the respective image sets. Color data were not employed in [11]. Considering the results in Table 4 and Table 6, we claim that our approach is competitive for WBC counting.

Hue as a feature

Although different studies employ color data to segment WBCs, the color is not processed as a feature, but the color components are processed separately. The WBCs are segmented by performing mathematical operations between the color components or in a specific color channel as if they were intensity channels; therefore, the chromaticity is not processed adequately because the hue components are uncorrelated. A major limitation is that the color channels are selected intuitively.

In [7], a color correction process using the L*a*b* color space was not automated. The red blood cells and background, using Otsu thresholding, and a combination of RGB, CMYK, and HSV color

space analysis were applied to segment WBCs. The noise was removed using a morphological filter and connected component labeling, and the circle Hough Transform was applied to detect overlapping cells. Di Ruberto et al. [8, 10] segmented WBCs with a support vector machine and used pixels taken from manually segmented ALL-IDB2 images to train the support vector machine. After the training phase, the classifier produced a labeled image with a different label for each image component.

Prinyakupt and Pluempitiwiriyawej [14] highlighted the WBC nucleus in the image by the sum of the R and B channels of the RGB space and divided the result by the value of channel G. The histogram equalization technique was applied, and the image was binarized with the Otsu method. The resulting image was used to segment the nucleus, and morphological operators were used to join the segmented nucleus with the lobes to remove noise and fill gaps. Liu et al. [17] located WBCs using multiple windows obtained by scoring multiscale cues to extract a rectangular region. The location window covered the entire WBC, and the segmented sub-images were obtained with a replacement procedure taken as initialization and the GrabCut algorithm.

Cao et al. [18] computed the dissimilarity measure between areas to cover all classes of the diameters of WBCs by applying a statistical analysis and K-means algorithm to color contrast (using L*a*b* space), edge density, gradient contrast, features based on color, canny edge, and gradient. These values were considered to set the size of the sliding windows. Zheng et al. [19] presented an unsupervised initial segmentation and supervised segmentation refinement. In the first stage, the overall foreground region was extracted from the cell image using the K-means algorithm, and then a coarse WBC region was generated and used to train a support vector machine classifier. This classifier classified each pixel of an image to improve segmentation. Median color features were introduced.

Cao et al. [21] considered the use of RGB and HSI color spaces for WBC count. They created images that were linear combinations of G, H, and S components that highlight the region of the

leukocyte nucleus. This approach was used for the segmentation of peripheral blood leukocytes. Hedge et al. [23] presented an algorithm for the classification of WBCs based on the features of the nuclei. The R, G, and B components of a color image were separated, and the R component was replaced with a contrast-enhanced G component to enrich the color of the nucleus. The segmentation of the image was performed using the TissueQuant method.

Mishra et al. [24] pre-processed the input images using the Y component of the CMYK image and a triangle method of thresholding. They employed a discrete orthonormal S-transform to extract the texture features to classify WBCs, and the dimensionality was reduced using linear discriminant analysis. Sudha and Geetha [25] mapped the input RGB image to the HSV image; from the S component, the borders were obtained using the gradient method, and the image was binarized by thresholding the gradient magnitudes. The border strength location windows were processed by describing the extent to which each pixel is connected to its neighborhood; pixels may have background and foreground nodes.

Table 7 summarizes the color spaces and color components employed in previous studies. In all of them, the color components are separated and intuitively treated in an essay - error manner.

Table 7. Color spaces and color components employed in related works.

Reference	Color spaces	Components employed	Cropped images
Safuan et al. [7]	RGB, CMYK, HSV, L*a*b*	S-G, C-G, S-C, H-Y; components L*, a*, and b* are used, separately, for image preprocessing.	No
Di Ruberto et al. [8, 10]	RGB	Statistical features computed per channel, separately.	No

Prinyakupt and Pluempitiwiriyawej [14]	RGB	G values divided by the sum of the average values of the R and B components.	No
Liu et al. [17]	L*a*b*	Histograms of each channel.	Yes
Cao et al. [18]	L*a*b*	Histograms of each channel.	Yes
Zheng et al. [19]	RGB, HSV	R, G, B average values H, S, V median values.	Yes
Cao et al. [21]	RGB, HSI	G, H, S processed separately.	Yes
Hedge et al. [23]	RGB	Channel R is substituted by channel G to form GGB.	Yes
Mishra et al. [24]	CMYK	Y	No
Sudha and Geetha [25]	HSV	S	No

In our proposal, chromaticity was processed without separating the hue components because the hue of the pixels was represented as a vector, preserving the correlation between the hue components. The vectors were processed using matrix operations. The staining and illumination conditions of the first 33 images of the ALL-IDB are different from the conditions of the last 75 images (Fig 7. Our proposal is robust for staining and illumination variations, and is competitive with respect to previous works (Table 4 and Table 6. Most of the studies have been developed to segment WBCs in cropped images; hence, such approaches may not be suitable for segmenting several WBCs within an image.

Furthermore, several of the methods listed in Table 7 employ clustering techniques that tend to create groups with the same size or number of elements for image segmentation. However, small parts within the image are not segmented successfully, and the performance of the clustering

techniques depends on the initial values of the centers. In addition, they require an a priori number of groups to group the data (pixels), but the number of groups depends on the image conditions.

Recent studies that used convolutional neural networks to segment WBCs [**Error! Reference source not found.-Error! Reference source not found.**] performed experiments with their private image databases, without the use of standard benchmarks, and their results cannot be compared with those of previous studies. The main contrast is that the computational load of convolutional neural networks is usually high, whereas the computational load of our implementation is low. Moreover, the size of the convolutional neural networks is fixed depending partially on the size of the input images. Owing to the different sizes of the images in the three image databases employed for experiments, the images must be resized to fit the size of the input layer of the convolutional neural network. The limitation of this approach is that the quality of the image may be degraded. In our approach, image resizing is not necessary.

Conclusions

We present a systematic formal method for leukocyte counting within blood smear images. The WBC segmentation is performed by selecting, via principal component analysis, pixels with a high chromatic variance that contrast with the average hue of the images. The best quantitative evaluation of this segmentation is obtained when the chromaticity is processed in the L*a*b* space. The segmentation performance is classified as excellent because the kappa index is greater than 0.8. Our method efficiently separates overlapping leukocytes by computing the intensity gradient and chromatic difference separately. The white blood cells were counted by applying connected component labeling to the binary version of the segmented image. Our proposal is competitive because the results of the quantitative evaluation and counting precision over the ALL-IDB benchmark

are similar in precision and sensitivity and better in specificity and accuracy than those of related studies. Furthermore, this method performs better because the color is processed as a proper feature, not decoupled in a trial-and-error experimental handling of components, as is done in most of the previous approaches. From the perspective of artificial intelligence, our method is bioinspired and mimics the human perception of color.

Acknowledgments

The authors wish to thank the Mexico National Council of Research (CONACyT in Spanish abbreviation), for the funding within the Basic Science Research Project A1-S-20037. We would like to thank Editage (www.editage.com) for English language editing.

Ethical approval

This paper does not contain any studies with animal or human subjects performed by any of the authors.

References

1. Tsai MH, Yu SS, Chan YK, Jen CC. Blood smear image based malaria parasite and infected-erythrocyte detection and segmentation. J Med Syst. 2015;39: 118.
2. Jan Z, Khan A, Sajjad M, Muhammad K, Rho S, Mehmood I. A review on automated diagnosis of malaria parasite in microscopic blood smears images. Multimed Tool Appl. 2018; 77: 9801-9826.

3. Abdulhay E, Mohammed MA, Ibrahim DA, Arunkumar N, Venkatraman V. Computer aided solution for automatic segmenting and measurements of blood leucocytes using static microscope images. J Med Syst. 2018;42(4): 58.
4. Mishra S, Majhi B, Sa PK. Texture feature based classification on microscopic blood smear for acute lymphoblastic leukemia detection. Biomed Signal Process Control. 2019;47: 303-311.
5. Liu H, Cao H, Song E. Bone marrow cells detection: a technique for microscopic image analysis. J Med Syst. 2019;43: 82.
6. Chung J, Ou X, Kulkarni RP, Yang C. Counting white blood cells from a blood smear using Fourier ptychographic microscopy. PloS ONE. 2015;10(7): e0133489.
7. Safuan SS, Tomari M, Zakaria W. White blood cell (WBC) counting analysis in blood smear images using various color segmentation methods. Measurement. 2018;116: 543-555.
8. Di Ruberto C, Loddo A, Putzu L. A leucocytes count system from blood smear images. Mach Vis Appl. 2016;27: 1151-1160.
9. Di Ruberto C, Loddo A, Putzu L. A multiple classifier learning by sampling system for white blood cells segmentation. Computer Analysis of Images and Patterns. CAIP 2015. Lecture Notes in Computer Science, vol 9257. 2015. pp. 415-425.
10. Di Ruberto C, Loddo A, Putzu L. Learning by sampling for white blood cells segmentation. Image Analysis and Processing — ICIAP 2015. Lecture Notes in Computer Science, vol 9279. 2015. pp. 557-567
11. Alomari Y, Abdullah SH, Azma RZ, Omar K. Automatic detection and quantification of WBCs and RBCs using iterative structured circle detection algorithm. Comput Math Method Med. 2014;2014(979302): 1-17.

12. Nazlibilek S, Karacor D, Ercan T, Sazli MH, Kalender O, Ege Y. Automatic segmentation, counting, size determination and classification of white blood cells. Measurement. 2014;55: 58-65.
13. Ghosh P, Bhattacharjee D, Nasipuri M. Blood smear analyzer for white blood cell counting: a hybrid microscope image analyzing technique. Appl Soft Comput. 2016;46: 629-638.
14. Prinyakupt J, Pluempitiwiriyawej C. Segmentation of white blood cells and comparison of cell morphology by linear and naïve Bayes classifiers. Biomed Eng Online. 2015;14: 63.
15. Cuevas E, Diaz M, Manzanares M, Zaldivar D, Perez-Cisneros M. An improved computer vision method for white blood cells detection. Comput Math Method Med. 2013;2013(137392): 1-14.
16. Dorini LB, Minetto R, Leite NJ. Semiautomatic white blood cell segmentation based on multiscale analysis. IEEE J Biomed Health Inform. 2013;17(1): 250-256.
17. Liu Y, Cao F, Zhao J, Chu J. Segmentation of white blood cells image using adaptive location and iteration. IEEE J Biomed Health Inform. 2017;21(6): 1644-1655.
18. Cao F, Liu Y, Huang Z, Chu J, Zhao J. Effective segmentations in white blood cell images using ε-SVR-based detection method. Neural Comput Appl. 2019;31: 6767-6780.
19. Zheng X, Wang Y, Wang G, Liu J. Fast and robust segmentation of white blood cell images by self-supervised learning. Micron. 2018;107: 55-71.
20. Sayed GI, Solyman M, Hassanien AE. A novel chaotic optimal foraging algorithm for unconstrained and constrained problems and its application in white blood cell segmentation. Neural Comput Appl. 2019;31: 7633-7664.
21. Cao H, Liu H, Song E. A novel algorithm for segmentation of leukocytes in peripherical blood. Biomed Signal Process Control. 2018;45: 10-21.
22. Liu H, Cao H, Song E. Bone marrow cells detection: a technique for microscopic image analysis. J Med Syst. 2019;43: 82.

23. Hedge RB, Prasad K, Hebbar H, Singh BK. Development of a robust algorithm for detection of nuclei and classification of white blood cells in peripherical blood smear images. J Med Syst. 2018;42(6): 110.

24. Mishra S, Majhi B, Sa PK. Texture feature based classification on microscopic blood smear for acute lymphoblastic leukemia detection. Biomed Signal Process Control. 2019;47: 303-311.

25. Sudha K, Geetha P. A novel approach for segmentation and counting of overlapped leukocytes in microscopic blood images. Biocybern Biomed Eng. 2020;40(2): 1-10.

26. Garcia-Lamont F, Cervantes J, Lopez A, Rodriguez L. Segmentation of images by color features: a survey. Neurocomputing. 2018;292: 1-27.

27. Janos A, Balazs F. Cluster analysis for data mining and system identification. Birkhäuser Verlag; 2007.

28. Landis R, Koch G. The measurement of observer agreement for categorical data. Biom. 1977;33(1): 159-174.

29. Shazad M, Umar AI, Khan M, Shirazi SH, Khan Z, Yousaf W. Robust method for semantic segmentation of whole-slide blood cell microscopic images. Comput Math Method Med. 2020;2020: 4015323.

30.

Figure1 Click here to access/download;Figure;Fig1.tif

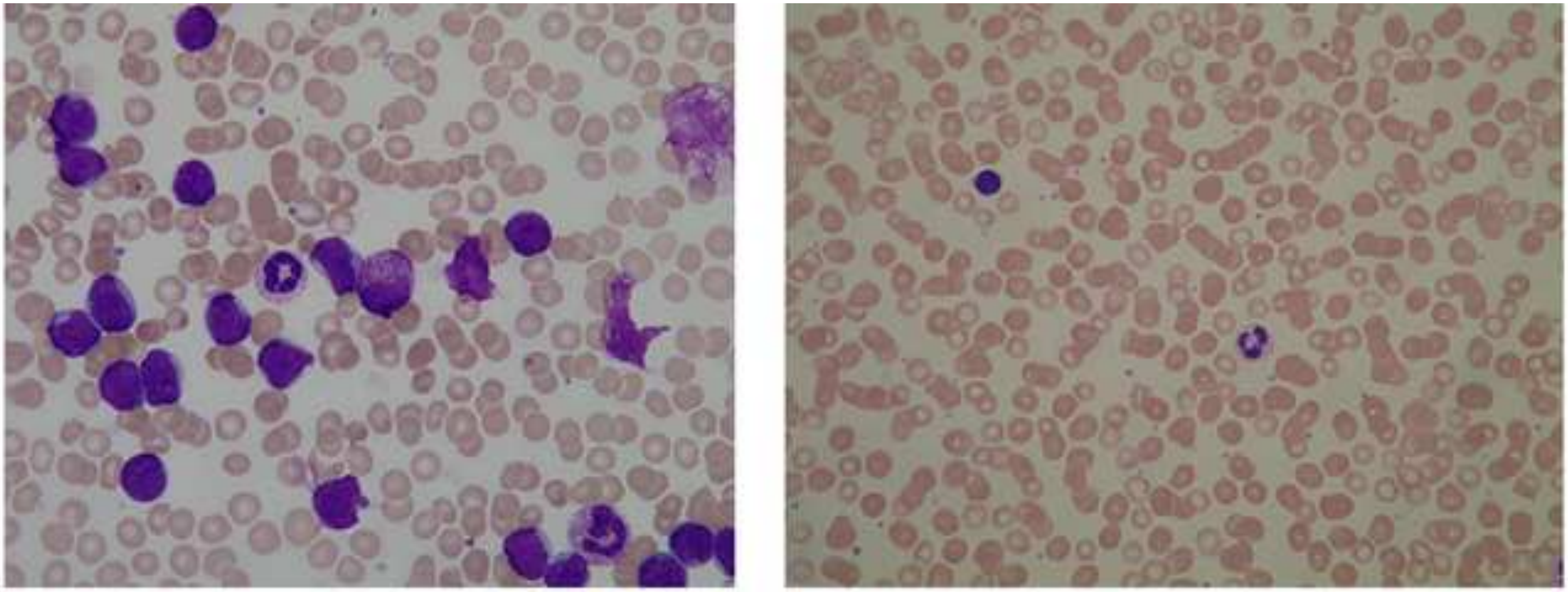

(a) (b)

Figure2

Click here to access/download;Figure;Fig2.tif

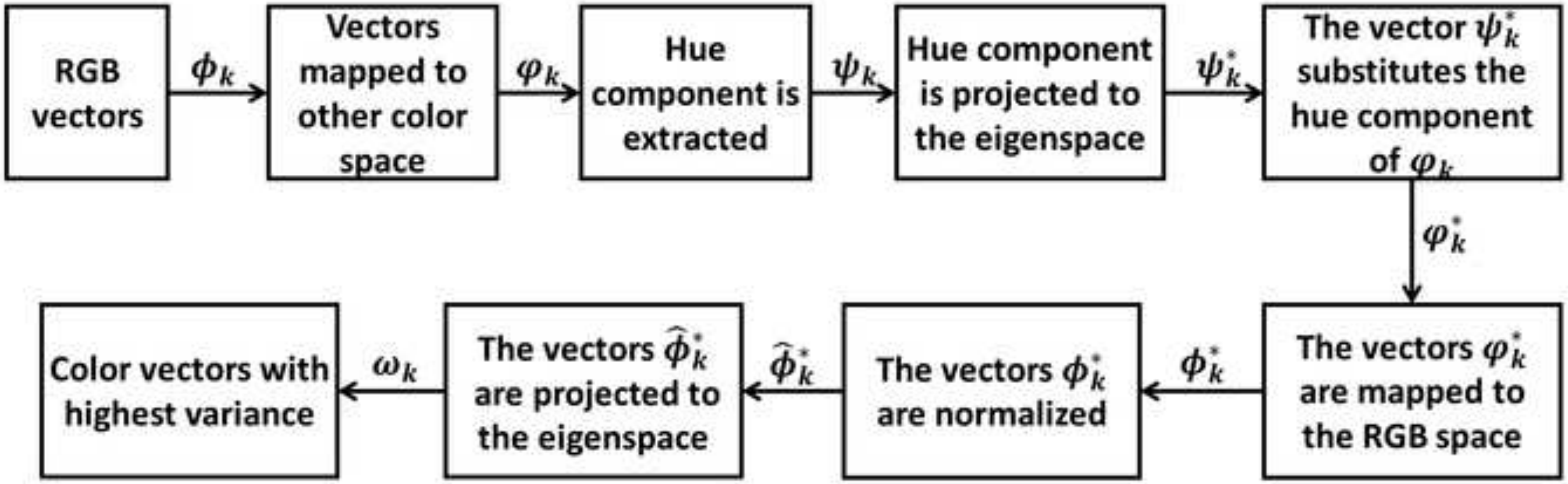

Figure3

Click here to access/download;Figure;Fig3.tif

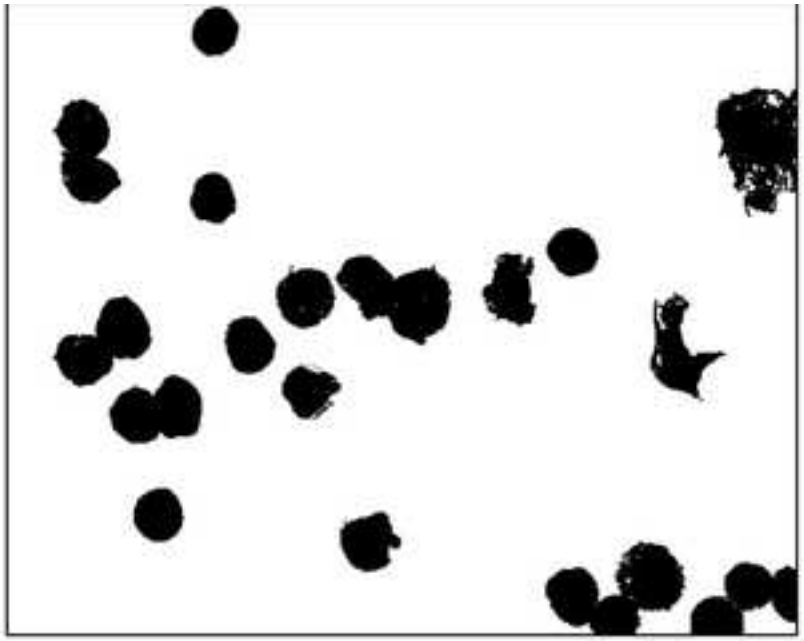

(a)

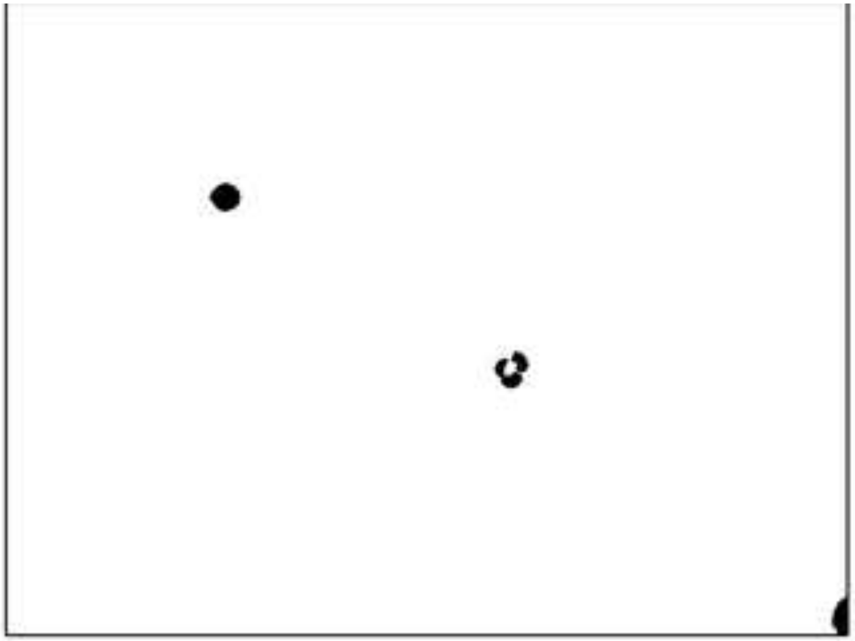

(b)

Click here to access/download;Figure;Fig4.tif

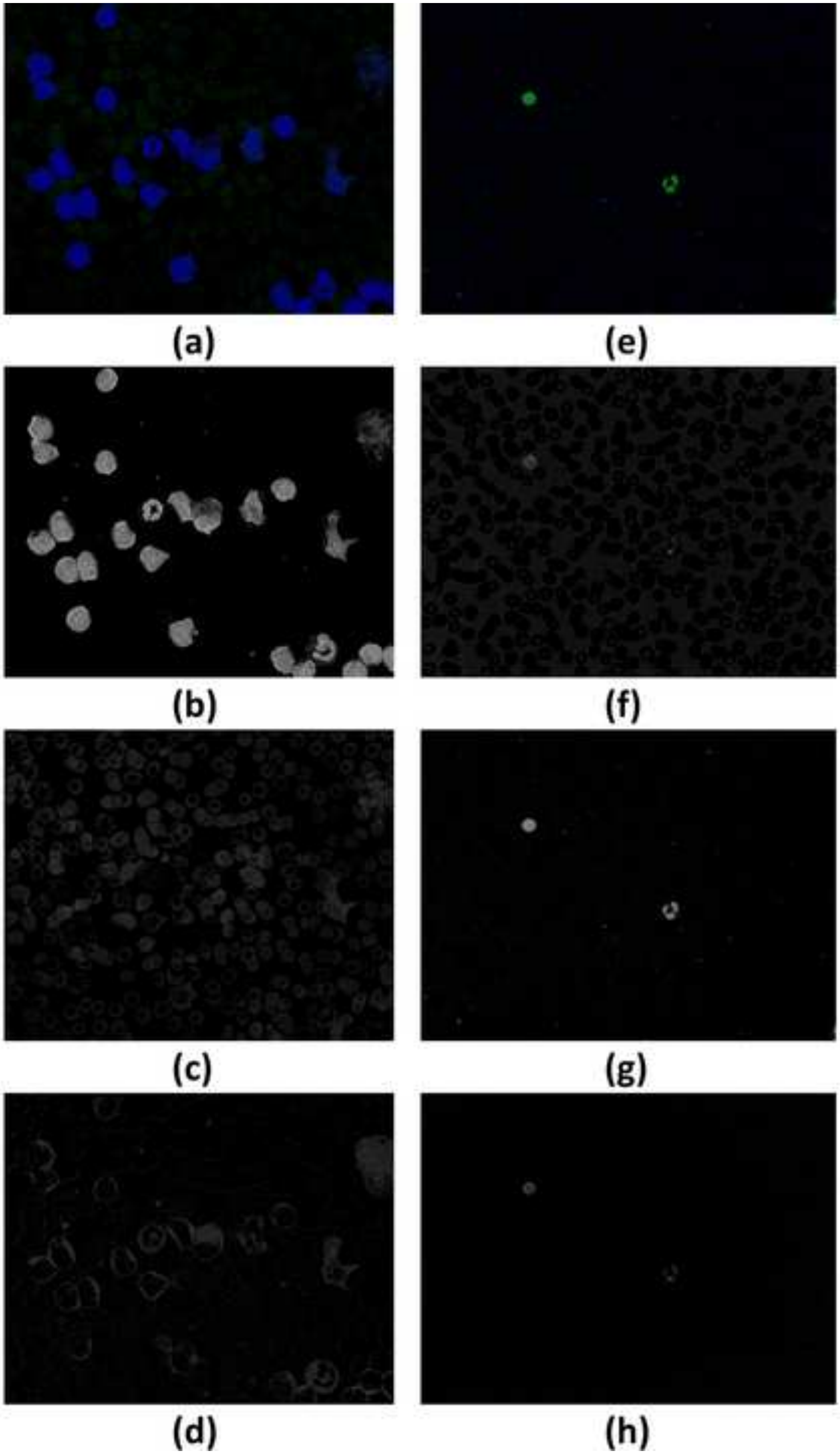

Figure5

Click here to access/download;Figure;Fig5.tif

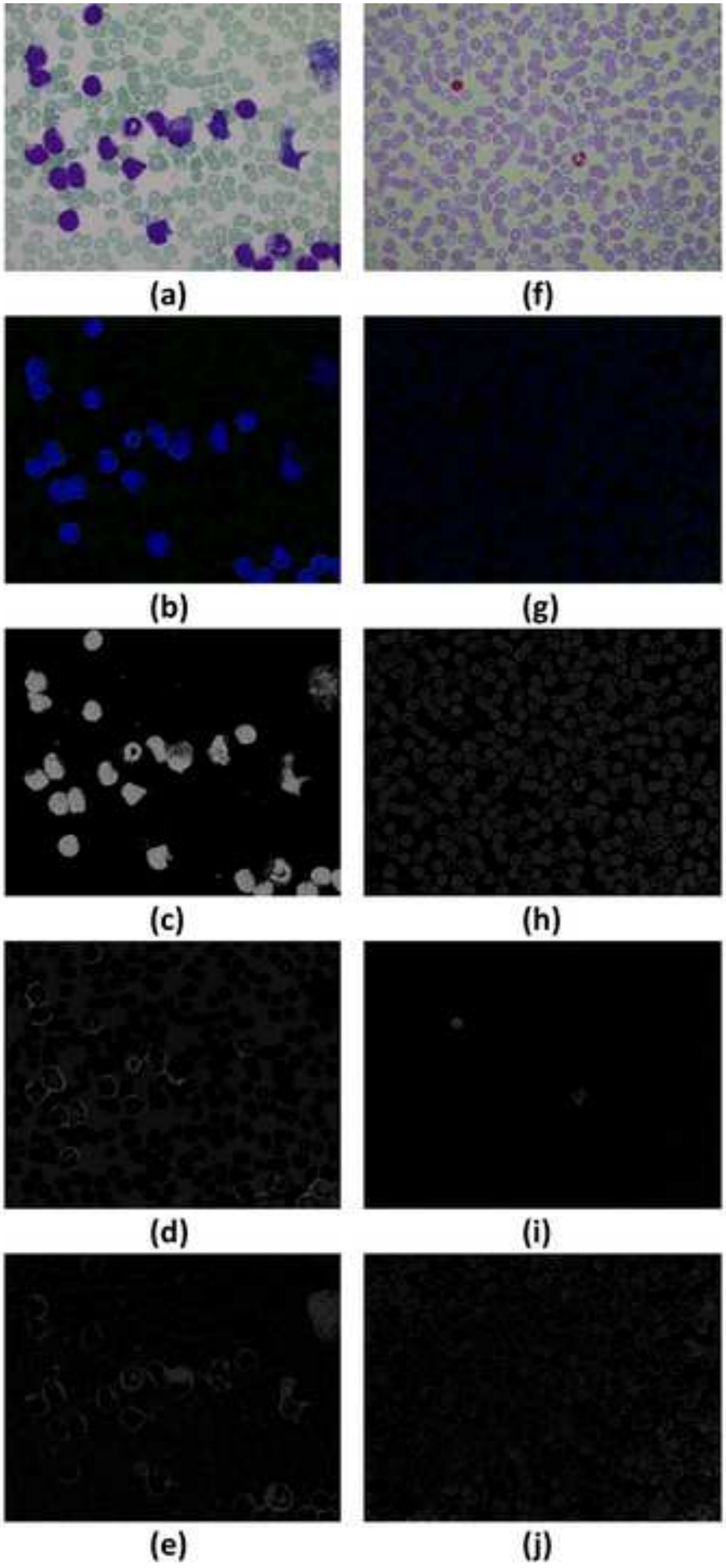

Figure6 Click here to access/download;Figure;Fig6.tif

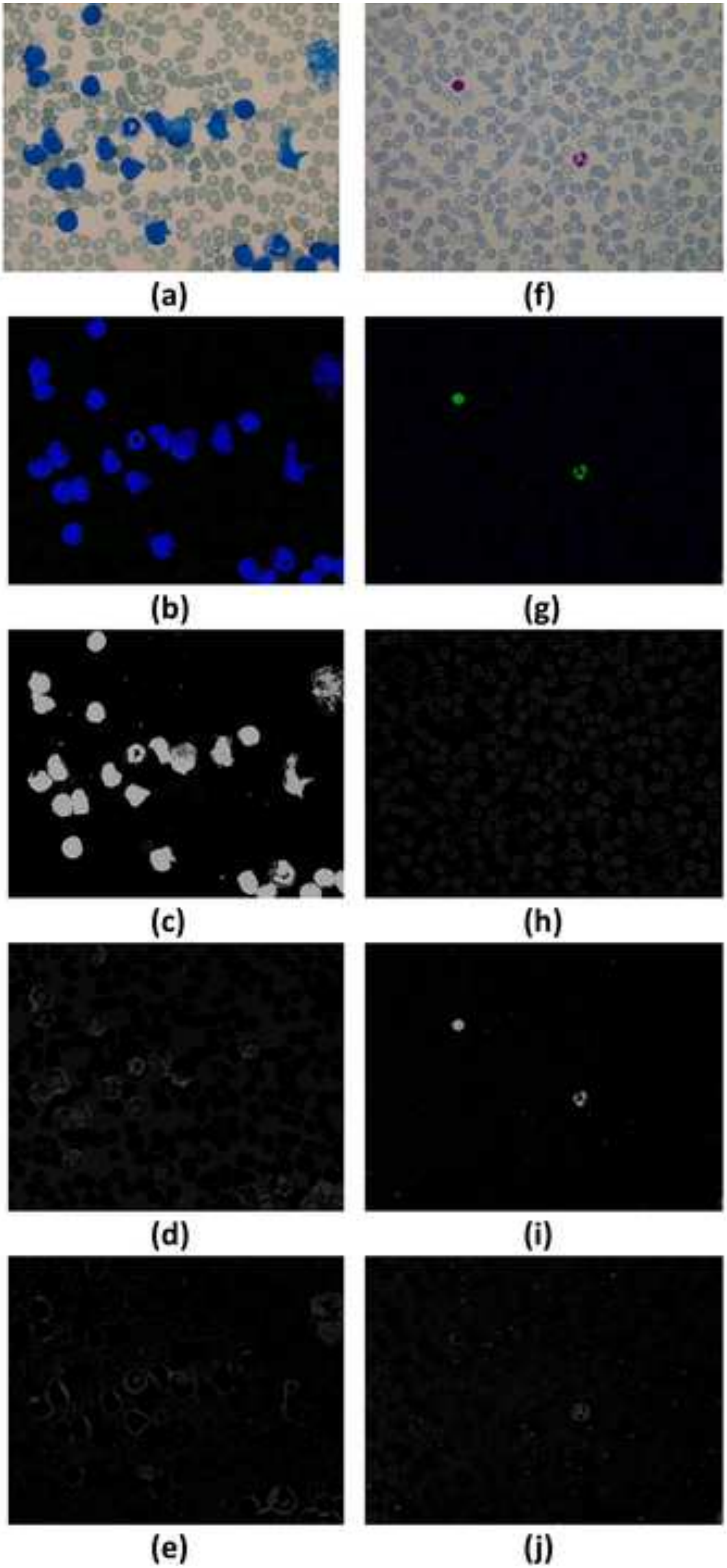

Figure7 Click here to access/download;Figure;Fig7.tif

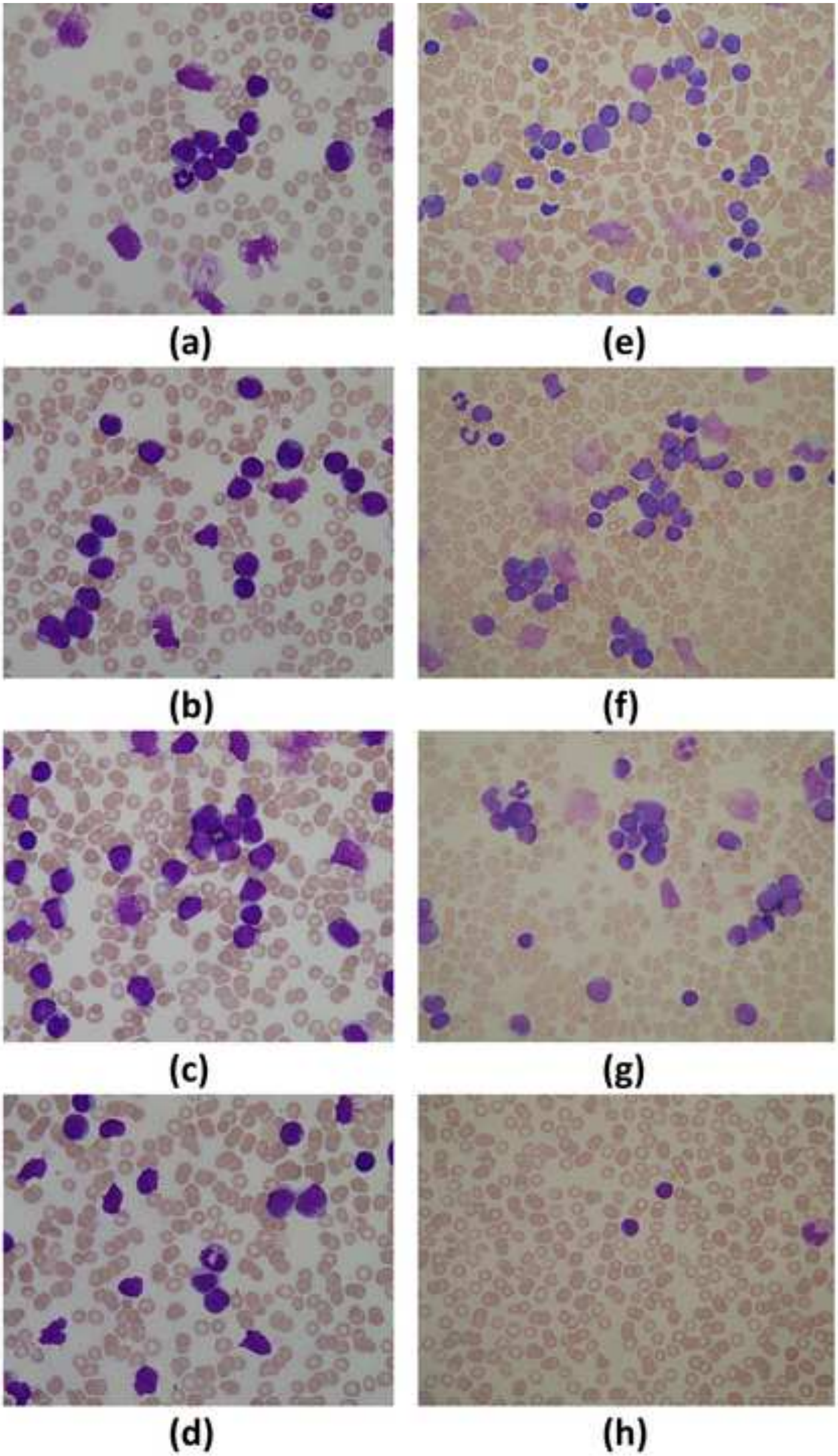

Click here to access/download;Figure;Fig8.tif

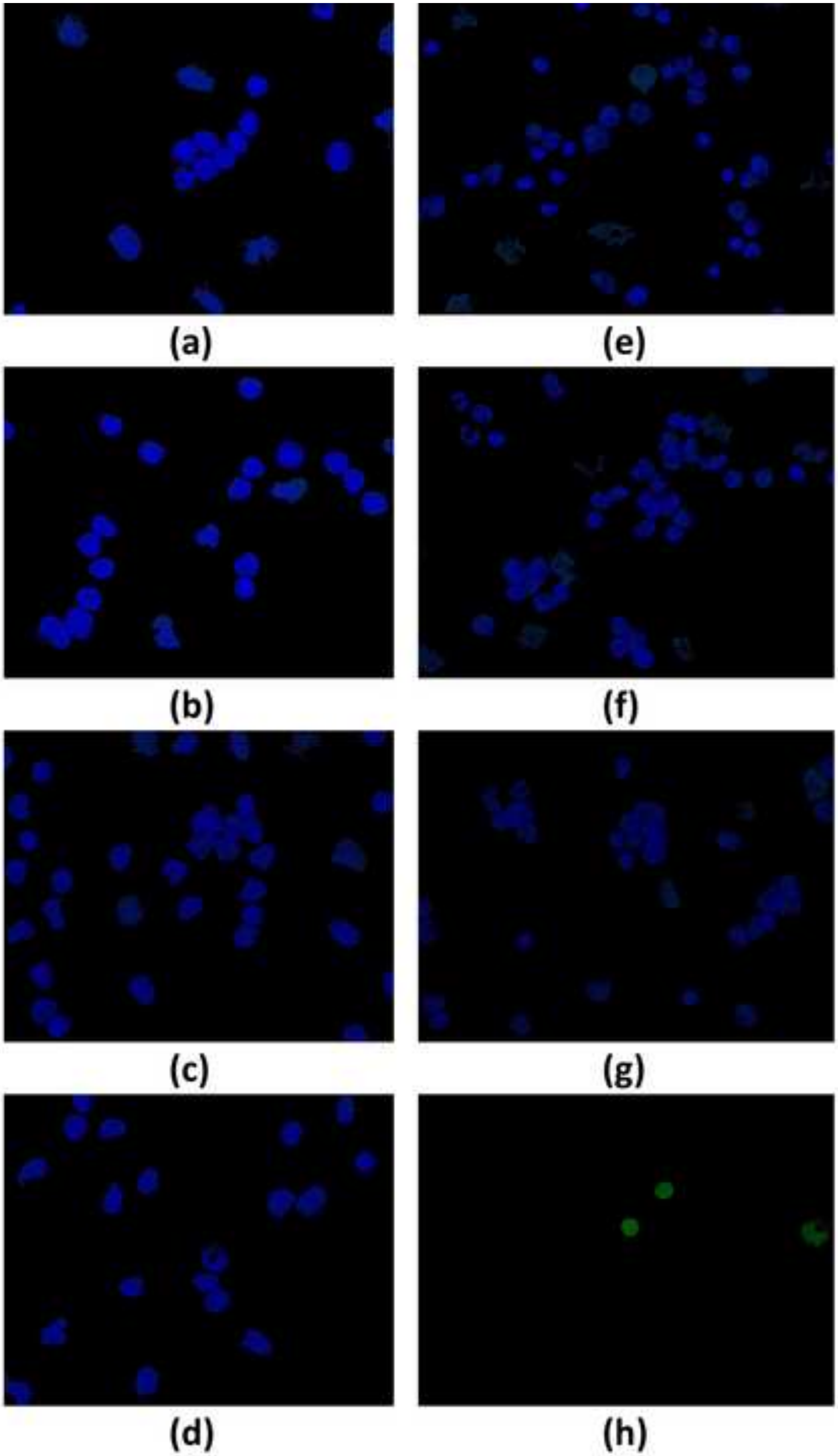

Figure9 Click here to access/download;Figure;Fig9.tif

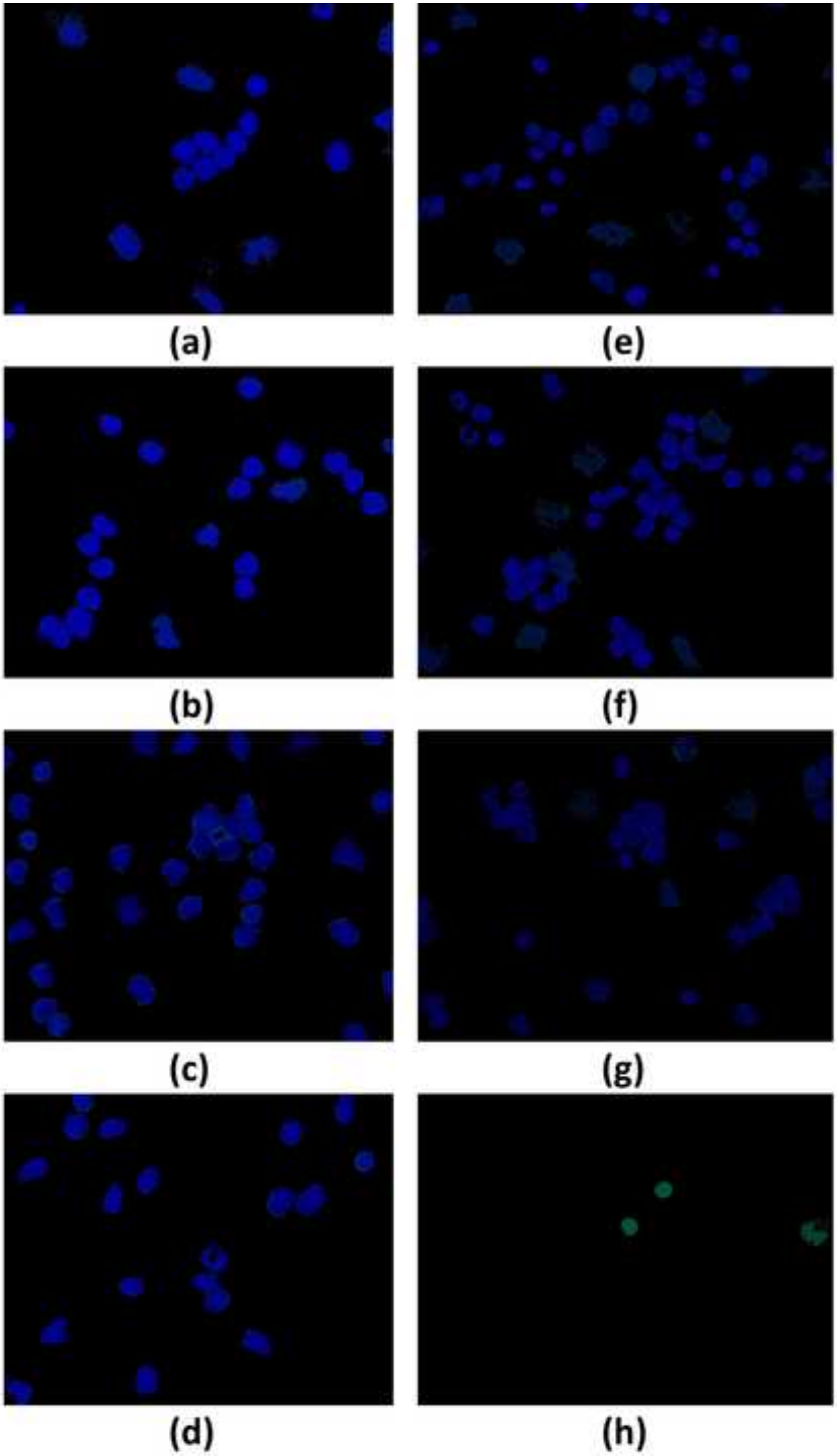

Figure10 Click here to access/download;Figure;Fig10.tif

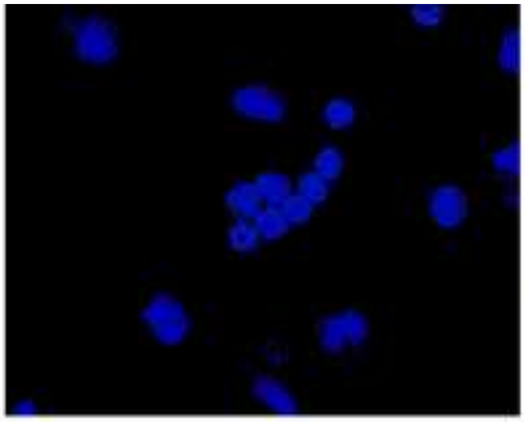

(a)

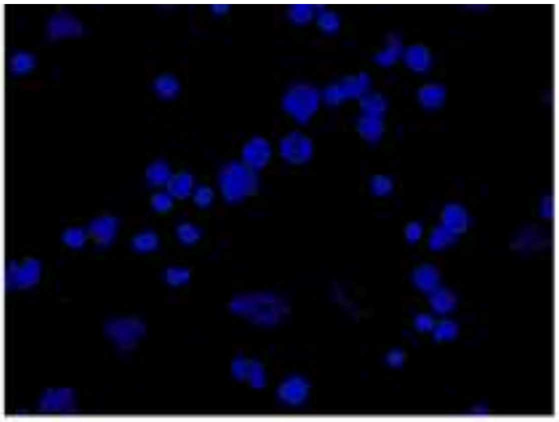

(e)

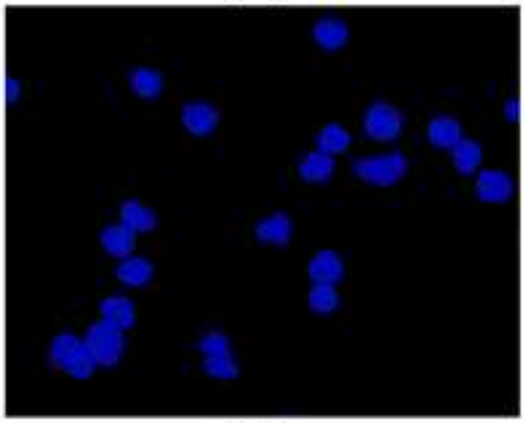

(b)

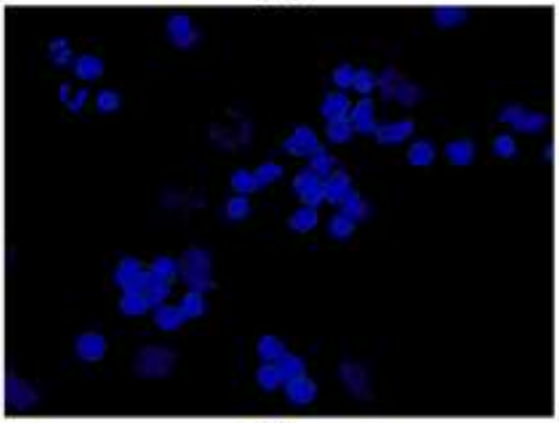

(f)

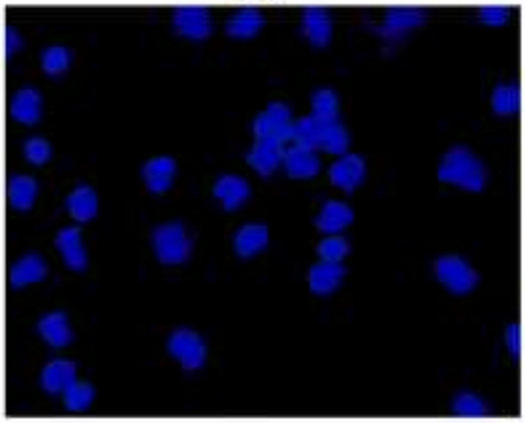

(c)

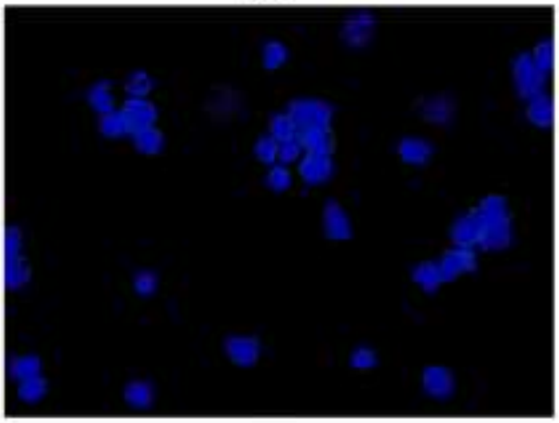

(g)

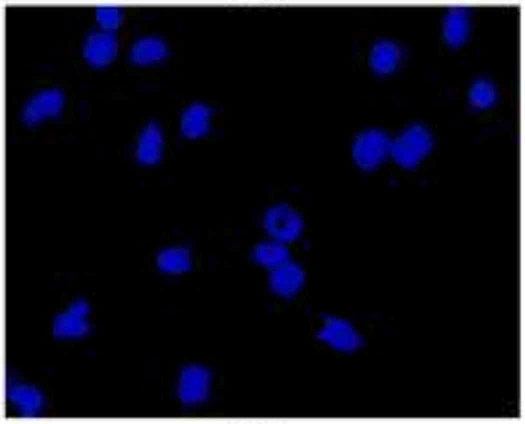

(d)

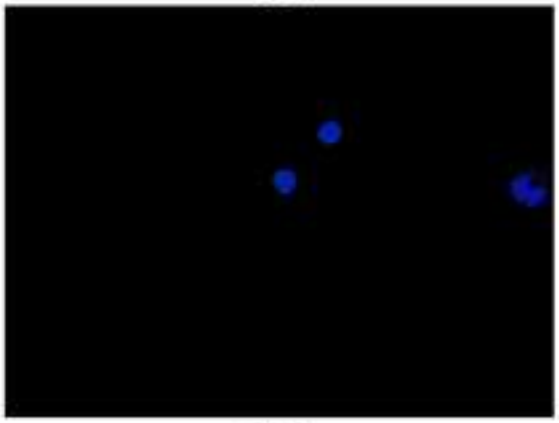

(h)

Script (matlab): Image processed in RGB space

Click here to access/download
Supporting Information
ImRGB.m

Script (matlab): Image processed in HSV space

Click here to access/download
Supporting Information
ImHSV.m

Script (matlab): Image processed in Lab space

Click here to access/download
Supporting Information
ImLab.m

Script (matlab)

Click here to access/download
Supporting Information
compare_segmentations.m

Click here to access/download
Supporting Information
Datos.xlsx

Copy of the manuscript highlighting the changes

Click here to access/download
Supporting Information
PCA_GarciaF_AlvaradoMatias_GarciaJ_PlosONE (1).docx

Certificate of editing

Click here to access/download
Supporting Information
Certificate_of_editing-VLLTW_1_zq8sw-h7et.pdf
