# Supplementary material for: Systematic segmentation method based on PCA of image hue features for white blood cell counting
Source: PLoS One. 2021 Dec 31;16(12):e0261857. doi: 10.1371/journal.pone.0261857 (PMC8719728; doi:10.1371/journal.pone.0261857)
Supplement: Attachment — Submitted filename: ResponsePlosOne.docx [file pone.0261857.s008.docx]

**Response to the reviewers’ comments**

**We really appreciate the reviewers’ comments. They were relevant and very useful to improve the presentation and organization of our research. Thank you so much.**

**Reviewer #1:** The authors present an algorithm to segment and count white blood cells using the principal components of the image hue features. The method seems interesting and relevant on the surface, and shows a performance within the range of the competitor methodologies they selected. The authors compared against recent methods, and explain their algorithm is distinguished because of they systematic approach to analysing colour relationships in the images. Also, they briefly describe an algorithm to segment overlapping cells.

… The authors are urged to check the uploaded PDF to see all the specific points I addressed in their manuscript…

**Response:**

We checked the points addressed in the mentioned PDF with the worth detailed suggestions by the reviewer Solis Lemus. We reorganized and fixed the writing of the paper attending these observations. We included the Section Previous Related Work; the Experiments and Results sections were separated. The named Methods and proposal section was renamed as Materials and methods. We really appreciate the neatness of this review.

**Reviewer #2:** The manuscript titled ‘Systematic segmentation method based on PCA of image hue features for white blood cell counting’ presents segmentation and counting of WBCs. In this work, chromaticity information in L*a*b* and HSV color spaces is extracted using PCA and used as a features for segmentation. Discussion on results and comparison is presented well.

*Major concerns:*

*1. No novelty. Principal component analysis (PCA), intensity gradient and chromatic difference are old approaches.*

**Response:**

PCA is a well-known technique. However, it has not been used to segment WBCs in blood smear images; mostly, different works focus to develop methods using shape features. Moreover, previous works that employ color data, they processed the color channels separately as if they were intensity channels. Other works perform mathematical operation between the color components, with the mathematical operations and the color components being intuitively selected. Our central remark is that processing the hue components separately, the correlation between them is lost; therefore, the chromaticity is not processed as a feature.

With our approach the chromaticity is processed as a feature, because the hue components are not processed separately, thus, the correlation between the hue components is kept.

We claim that a person can locate the WBCs by the chromatic difference regarding the other elements of the image. This approach has not been addressed, according to the related works we have reviewed. By using PCA the human perception is emulated because the colors (data) are mapped such that the chromatic variance of the pixels is maximized, thereby the WBCs can be segmented.

*2. Poor literature survey.*

**Response:**

We now included the Section of **Related works**, to enough review the methods through the previous works.

*3. Is it segmentation problem or classification problem? The parameters estimated are used for classification.*

**Response:**

The contribution of this study is on the segmentation and counting of white blood cells. The parameters estimated are employed for segmentation. We do not classify white blood cells.

*4. Incomplete details about methodology used for WBCs segmentation, overlapping WBCs separation.*

**Response:**

We have revised, extended, and improved the explanation of our methodology. Please, see the Section **Material and Methods**.

*5. Evaluation of automated segmentation may be done with dice similarity coefficient (DSC), Jaccard similarity coefficient also.*

**Response:**

The dice similarity coefficient is also known as f-score. The results using this metric are presented in the Section of **Results**. As the reviewer suggests, we have evaluated our resulting images using the Jaccard similarity coefficient, and the results are presented in the Section of **Results**.

*6. Visualization of segmented results should be compared with corresponding ground truth images. It should be presented in manuscript.*

**Response:**

We have included the ground truth images to compare the resulting images shown in figure 8, 9 and 10. Please, see them in Section XX.

*Minor Suggestions*:

*Response.*

**We appreciate and thank these minor but important corrections indicated by the reviewer.**

*1. Fig. 1: more details regarding blood smear images with size, camera and illumination should be illustrated.*

**Response:**

We added more details to describe the differences between the images of figure 1.

*2. The remainder of the paper is organized as follows …. > all sections are numbered as ‘section 0’.*

**Response:**

We corrected the paragraph.

*3. Fig.2 needs further explanation and better illustration for readers’ understanding perspective.*

**Response:**

We have rewritten the explanation of figure 2.

*4. Equations are not cited in text.*

**Response:**

We cited all the equation in the text.

*5. ….processing the hue in the RGB space of the images…. How do you get hue in RGB image?*

**Response:**

The colors in the RGB space are represented as three-element vectors. The intensity and the hue of the colors are represented by the magnitude and the orientation of the vectors, respectively. Hence, the vectors are normalized, thereby the chromaticity can be processed.

*6. Explanation of figures (Fig. 7-10) should be elaborated.*

**Response:**

We have included two paragraphs with those figures explained.

*7. … M is the total number of detected WBCs…. It may be only WBCs and not detected WBCs.*

**Response:**

This formula is extracted from the reference [31].

*8. Do check the error in citation ….Recent studies that used convolutional neural networks to segment WBCs [Error! Reference source not found.-Error! Reference source not found.]*

**Response:**

We corrected the cross reference.
