# Supplementary material for: Systematic segmentation method based on PCA of image hue features for white blood cell counting
Source: PLoS One. 2021 Dec 31;16(12):e0261857. doi: 10.1371/journal.pone.0261857 (PMC8719728; doi:10.1371/journal.pone.0261857)
Supplement: Attachment — Submitted filename: Rebutal3.docx [file pone.0261857.s009.docx]

# **Response to the reviewer**

We appreciate the comments of the reviewer and for taking the time to review the paper. Next, we give response to his comments.

**Reviewer #1:** *I want to acknowledge that the authors have put a substantial amount of work into this version of the manuscript. Many aspects have improved from the first time I reviewed it.
I think some things are still slightly out of place, but overall the quality of the manuscript has improved.*

**Reviewer #1:**

*The authors' work improves on accuracy and specificity compared to other works, however the counting precision is still below their competitors' and no comment is provided on this. Perhaps the authors' method is more versatile for a wider range of smear images, or perhaps it is quicker, or has a greater potential for expansion and improvement? Or perhaps mention what is it in the others' works that cause them those higher precision numbers? Maybe it is a problem with the overlapping solution proposed? There's a lot of questions that could be addressed.*

**Response:**
We included the paragraphs (lines 327-342) below the table 6 (Segmentation and counting Section) and the two paragraphs (lines 392-406) of the Discussion section, to give response to the reviewer’s comments.

**Reviewer #1:**

*Add a comment on the overlapping separation procedure. I suspect therein lie the problems of counting, but there is little to be said other than the code provided and the explanation in the manuscript.*

**Response:**
We give response to this comment in the first paragraph (lines 327-331) below the table 6 (Segmentation and counting Section).

**Reviewer #1:**

*Please check the English writing throughout the manuscript. For example, in the sentence starting in "Often," in lines 32-34.*

**Response:**

We checked and reviewed the English writing of the manuscript.

**Other minor corrections:**

**Reviewer #1:**
*- Move the explanations on lines 301-305, and 308-310 to the Quantitative Evaluation section.*

**Response:**
We moved the lines mentioned by the reviewer to the Quantitative Evaluation section.

**Reviewer #1:**

*- Move the first and last sentence of the paragraph in lines 326-330 to the Discussion. Maybe elaborate on this point, and provide some more information. I would like to see something like the average computing time per image reported. Maybe this is an area where the authors' work is superior and might be noteworthy.*

**Response:**
We moved the sentence mentioned by the reviewer to the Discussion section. In the paragraphs between lines 392-406, we give response to the reviewer’s comments.

**Reviewer #1:**

*- Add future work to this pipeline.*

**Response:**

In the last paragraph’s line of the Discussion section, we give response to the reviewer’s comment (line 411).
